# Artificial intelligence and machine learning for hemorrhagic trauma care

**DOI:** 10.1186/s40779-023-00444-0

**Published:** 2023-02-16

**Authors:** Henry T. Peng, M. Musaab Siddiqui, Shawn G. Rhind, Jing Zhang, Luis Teodoro da Luz, Andrew Beckett

**Affiliations:** 1 Defence Research and Development Canada, Toronto Research Centre, Toronto, ON M3K 2C9 Canada; 2 Sunnybrook Health Sciences Centre, Toronto, ON M4N 3M5 Canada; 3 St. Michael’s Hospital, Toronto, ON M5B 1W8 Canada; 4 Royal Canadian Medical Services, Ottawa, K1A 0K2 Canada

**Keywords:** Artificial intelligence, Hemorrhage, Machine learning, Trauma, Injury

## Abstract

Artificial intelligence (AI), a branch of machine learning (ML) has been increasingly employed in the research of trauma in various aspects. Hemorrhage is the most common cause of trauma-related death. To better elucidate the current role of AI and contribute to future development of ML in trauma care, we conducted a review focused on the use of ML in the diagnosis or treatment strategy of traumatic hemorrhage. A literature search was carried out on PubMed and Google scholar. Titles and abstracts were screened and, if deemed appropriate, the full articles were reviewed. We included 89 studies in the review. These studies could be grouped into five areas: (1) prediction of outcomes; (2) risk assessment and injury severity for triage; (3) prediction of transfusions; (4) detection of hemorrhage; and (5) prediction of coagulopathy. Performance analysis of ML in comparison with current standards for trauma care showed that most studies demonstrated the benefits of ML models. However, most studies were retrospective, focused on prediction of mortality, and development of patient outcome scoring systems. Few studies performed model assessment via test datasets obtained from different sources. Prediction models for transfusions and coagulopathy have been developed, but none is in widespread use. AI-enabled ML-driven technology is becoming integral part of the whole course of trauma care. Comparison and application of ML algorithms using different datasets from initial training, testing and validation in prospective and randomized controlled trials are warranted for provision of decision support for individualized patient care as far forward as possible.

**Supplementary Information:**

The online version contains supplementary material available at 10.1186/s40779-023-00444-0.

## Background

Trauma is a major global public health issue, causing nearly 6 million deaths worldwide each year [1]. Even with significant advances in trauma care, especially through a comprehensive damage control strategy, traumatic injury remains the leading cause of death worldwide in people aged 18–39 years. Most of these deaths are represented by hemorrhage with one-half of them happening in the pre-hospital setting [2, 3]. Uncontrolled hemorrhage complicated by trauma-induced coagulopathy is also the major cause of death on the battlefield [4, 5]. Moreover, multi-domain operations are expected in foreseeable combat, where prolonged field care becomes more frequent when air superiority is yet to be assured [6]. Future combat operations anticipate delayed evacuation, prolonged and more complex field care, and potential for clinical complications.

In the past few years, artificial intelligence (AI) has drawn tremendous attention for its potentials for utility in every facet of human activities including health care [7]. AI is primarily a computer science concept where a computer system simulates human intelligence, including speech recognition, predictive modeling, and problem solving [8].

Machine learning (ML), considered the primary means to achieve AI, is to provide statistical/modelling rules to a computer system for it to gain information from data (i.e., learn), without explicit human programming. ML has been increasingly used for data analyses (e.g., learning to explain processes), and gain additional knowledge from data (e.g., prediction of outcomes). The ML approaches have recently gained popularity in medicine because of their ability to improve modelling algorithms autonomously. In particular, ML has shown promising results in medical services and medical emergencies, positively impacting areas including pre-hospital care and disease screening, clinical decisions, and mobile health [9].

AI has already been used in research and clinical settings, with extensive research going into the use of AI and ML for cancer diagnosis and therapy, or for use in precision medicine and/or drug discovery [10–12]. A body of review articles have recently emerged that showcase the use of AI in trauma/emergency care. For example, the potential of AI on the prediction of trauma volume or acuity irrespective of the center capacity has been observed, creating room for optimized resource allocation and improved patient care [13]. Similarly, AI-enabled precision medicine in trauma has been reviewed [14]. The framework for AI research in combat casualty care has also been developed [15]. However, the topic on ML for hemorrhagic trauma care has not been comprehensively reviewed.

Given the capability of ML in extracting important features from large multidimensional data sets predicting real-life outcomes, it is often seen as having significant potential in the field of trauma when it comes to improving access and quality of care, across different regional trauma systems and within a local trauma environment [16]. Trauma incorporates numerous factors in many forms affecting different organs, and their consequence could be related to the individual’s physiological attributes (e.g., age, fragility, premedical conditions) [17]. These factors translate into substantial quantity of data features, leading to high dimensional data. As such, if only with traditional mathematical modelling methods, quantifying its effects on individuals is challenging.

Therefore, to better elucidate the current role of AI in trauma care and contribute to the future development of ML, we conducted a literature review on AI with a focus on ML for the management of traumatic hemorrhage. The paper aims to review the advancements and new approaches that are being implemented in assessment of risk given a severe injury, prediction and/or resource allocation for transfusion, hemorrhaging and coagulopathy and prediction of patient disposition following hospital arrival. These advancements could be useful in the development of AI solutions that will provide expeditious decision-making for front line staff providers in urgent care in the said areas. To achieve this, we aimed to provide an overarching narrative of AI and its use in addressing patient care in various facets of trauma care.

## Search strategy, selection and inclusion criteria

A search in PubMed (January 1, 1946–January 14, 2022) and in Google scholar (first 100 hits) were carried out restricting to English-language articles using the following keywords: “artificial intelligence” or “machine learning” and “trauma*” in combination with one of the following: “bleeding”, “care”, “coagulopathy”, “hemorrhage” or “haemorrhage”, “mortality”, “military”, “outcome”, “resuscitation”, “shock”, “soldiers”, “triage”, “transfusion” as well as using the combinations of “artificial intelligence” or “machine learning” and “combat casualty care”. A full search strategy and combination of keywords used can be viewed in Additional file 1: Table S1.

Titles and abstracts were screened independently to determine relevance and, if deemed appropriate, the full article was reviewed. Additional publications were selected from the cross-references listed in the included original papers and from the cited articles. Disagreements were resolved by consensus or with another review author. The same strategy was used for data extraction and analyses as described later. The screening, full text review, and extraction were conducted online using Covidence (Veritas Health Innovation Ltd., Melbourne, VIC, Australia) [18].

Studies were eligible if they examined AI/ML for the prediction, management, and treatment requirements of traumatic hemorrhage. The review focused on human studies conducted in trauma patients with severe bleeding. It should be noted that animal models play important roles in traumatic hemorrhage and resuscitation research [19, 20] and AI/ML techniques have been applied in animal models of hemorrhage [21–23], which deserves further investigation. Studies in burns were excluded given a recent review on this topic [24]. We also excluded studies in other types of injuries if patients did not present with severe bleeding. Review articles were excluded unless they were focused on or directly related to hemorrhagic trauma. Papers related to AI in trauma surveillance, systems optimization, education, and training were also excluded.

Data were abstracted from all studies using a standardized form consisting of article title, authors, year, study aims/objectives (prediction of trauma outcomes, risk assessment and injury severity, prediction of coagulopathy, detection of hemorrhage, and transfusion requirements), study design (retrospective or prospective observational cohort or case-control studies), study population (size, database, inclusion and exclusion criteria), model development including methodology, relevant features, various algorithms, model performance and validation. In addition, the frequencies of ML algorithms, features, databases, and sample sizes were summarized. We also conducted comparisons of performance between different ML-assisted trauma care and standard of care within and across different studies for further insight into validation approaches and future work. The overall benefits and limitations of ML on trauma care were also discussed.

Different metrics have been used to measure the performance of ML algorithms. We used area under receiver operating characteristic (AUROC) curve, accuracy, precision (positive predictive values), sensitivity, specificity and *F*-value as extracted from the original studies for comparative analyses. The AUROC has been defined and used to compare prediction performance of ML-based models for various applications. A model with an AUROC of 1.0 is a perfect discriminator and is an indicator that a model is able to perfectly distinguish between all the positive and the negative class points correctly. Furthermore, 0.90–0.99 is considered excellent, 0.80–0.89 is good, 0.70–0.79 is fair, and 0.51–0.69 is considered poor/not statistically significant [25].

## Application of ML algorithms for hemorrhagic trauma

The last decade has seen huge leaps in computation performance and accessibility of ML methodology, along with access to growing digitalized information and datasets. This review highlighted an increasing interest in the application of ML to various trauma research settings. Since AI in trauma care is still an emerging field, inclusion categories of references synonymous with this topic aim to provide a thorough understanding of current research in this field. Studies classified under risk assessment and trauma outcome are the two largest categories of studies included in this study and involve the use of datasets similar to the other categories. The understanding of models developed in these studies may provide insight into the multi-faceted applications that these similar datasets may offer in different objectives. The ML models included in the review have demonstrated capability through achieving high performance, which may translate in their use for diagnosing, predicting, and prognosticating in severe bleeding injured trauma patients. In addition, the models could play a significant role in evaluating the quality of care delivered by healthcare systems, optimize vital resource management in hospitals and remote settings, and offer decision-support tools to ensure efficient care.

**Fig. 1:**
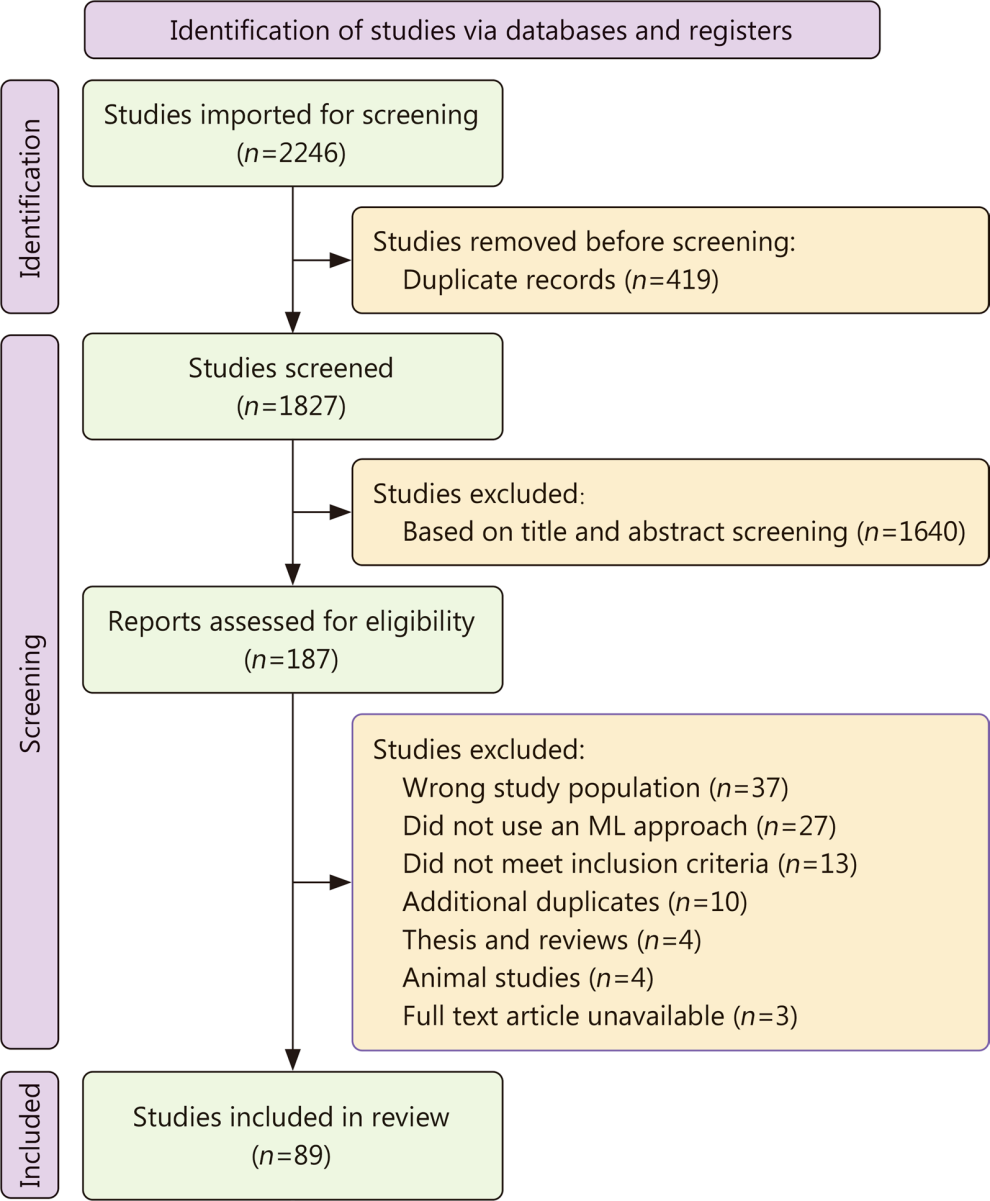
Flow chart of study selection

For this review, a total of 1827 studies were imported through the search from the two databases (Fig. 1). Initial title and abstract screening yielded 187 studies, and once fully reviewed in terms of the inclusion criteria, 89 studies were included, with their content analysed and discussed. Thirty-seven studies were excluded as they did not involve patients who suffered hemorrhagic trauma, 27 fell under the study exclusion criteria (study population that included patients with burns, musculoskeletal injuries, pulmonary injuries, wound infections, in vitro and animal studies/models, papers concerning surveys, opinions, ethics and policy of AI for traumatic health care), 13 did not use an ML approach, and 21 studies were excluded for other reasons (full text article unavailable, animal studies, review papers, additional duplicates found along data extraction).

Henceforth, categories were identified through literature. This classification subjected various study topics into following general categories, with priority focus on the application of ML algorithms for hemorrhagic trauma: (1) outcome prediction (mostly discharge/mortality); (2) risk assessment and injury severity for triaging; (3) prediction for transfusion and/or transfusion requirements; (4) detection of hemorrhage; (5) prediction of coagulopathy. The category identified more frequently was prediction of the outcome of trauma (*n* = 45), followed by risk assessment and injury severity for triaging (*n* = 18), transfusion prediction (*n* = 11), detection of hemorrhage (*n* = 11), and finally prediction of coagulopathy (*n* = 4). Additionally, a review surveying the various ML algorithms in trauma [26]. A summary of some of these results can be found in Table 1, while a full summary of the study design, ML models utilized, and performance of the models of all the studies included in this review is presented in Additional file 1: Table S2.

**Table 1:** Summary of main studies included in the review (see Additional file 1: Table S2 for full study summaries)

Authors	Year	Purpose	Methodology	Features	Dataset used	Dataset size	AUROC (or relevant performance metric)
Ahmed et al.[27]	2020	Mortality prediction model	DNN	Age, INR, PT, PTT, haemoglobin, hematocrit, WBC, platelets, creatinine, glucose, lactate	MIMIC III	3041	AUROC: 0.912
Kilic et al. [28]	2010	Determining time period for calculation and evaluation of trauma severity and predicted mortality after a period of resuscitation	Fuzzy-logic inference system	SBP, GCS, changes after 1 h of resuscitation	Data from hospital/ER records	150	AUROC: 0.925
Kuo et al. [29]	2018	Mortality prediction of motorcycle riders suffering traumatic injuries	SVM	Age, SBP, HR, RR, RBC, platelet, haemoglobin, hematocrit, GCS, AIS, ISS	Data from hospital/ER records	946	AUROC: 0.9532
Maurer et al. [30] and El Hechi et al. [31]	2021	Trauma-outcome predictor (TOP) smartphone tool	TOP	Age, SBP, HR, RR, SpO_2_, Temperature, comorbidities, GCS, injury mechanism, AIS	ACS-TQIP	934,053	AUROC (penetrating trauma: 0.920, blunt trauma: 0.830)
Cardosi et al. [32]	2021	Predicting trauma patient mortality	XGBoost	Age, SpO_2_, PR, RR, Temperature, GCS, injury type	NTDB	2,007,485	AUROC (children data: 0.910, adult data: 0.890, all aged data: 0.900)
Lee et al. [33]	2021	Prognostic prediction for critical decision-making	XGBoost	Age, HR, RR, MAP, GCS, AIS	Data from hospital/ER records	2232	AUROC: 0.940
Tran et al. [34]	2021	Mortality prediction model	XGBoost	Injury mechanism	NTDB	1,611,063	AUROC: 0.863
Tsiklidis et al. [35]	2020	Outcome predictor for survival	Gradient Boost	Age, SBP, HR, RR, Temperature, SpO_2_, GCS	NTDB	799,233	AUROC: 0.924
Becalick et al. [36]	2001	Assessing probability of survival after trauma	ANN	Age, RR, SBP, SpO_2_, HR, Injury type, AIS, ISS, GCS	UKTARN	2042	AUROC: 0.921
Sefrioui et al. [37]	2017	Predicting patient survival using readily available variables	SVM	Age, injury type, BP, GCS, RR,	NTDB	656,092	AUROC: 0.931
Batchinsky et al. [38]	2009	Predicting life-saving intervention based on EKG derived data	ANN	Heart rate complexity	USAISR Trauma	262	AUROC: 0.868
Liu et al. [39]	2017	Predicting life-saving intervention	MLP	HR, SBP, DBP, MAP, RR, SpO_2_, SI, PR	WVSM trial	79	AUROC: 0.990
Liu et al. [40]	2018	Predicting life-saving intervention	MLP	HR, SBP, DBP, MAP, RR, SpO_2_, SI, PR	WVSM trial	104	Correlation coefficient: 0.779
Kim et al. [41, 42]	2018, 2021	Decision-making algorithm for remote triaging	DNN	Age, HR, SBP, SI, SCS	NTDB	1,204,290	AUROC: 0.890
Scerbo et al. [43]	2014	ML model for triaging trauma patients	RF	Age, HR, SBP, DBP, SpO_2_, RR, GCS, injury type	Data from hospital/ER records	1653	Sensitivity: 0.890, Specificity: 0.420
Nederpelt et al. [44]	2021	In-field triage tool for determining shock, MT, need for major surgery	Dirichlet DNN	Age, BMI, HR, SBP, RR, Temperature, GCS, injury location	ACS-TQIP	29,816	AUROC (shock: 0.890, MT: 0.860, need for major surgery: 0.820)
Follin et al. [45]	2016	Predicting need for specialized trauma care	DT	Age, HR, SpO_2_, SBP, GCS, ISS, injury mechanism	Data from anonymized prospective trauma registry	1160	AUROC: 0.820
Mina et al. [46] and Hodgman et al. [47]	2013, 2018	Smartphone app for predicting Massive Transfusion cases	LASSO regression	Mechanism of injury, HR, SBP, BD, ISS, RBC, resuscitation intensity	Data from hospital/ER records. Validation data from PROMMTT database	10,900/1245	AUROC (training: 0.956, validation: 0.711)
Feng et al. [48]	2021	Demand prediction for traumatic blood transfusion	XGBoost	Trauma location, Age, HR, RR, SI, SBP, DBP, SpO_2_, Temperature	Data from hospital/ER records	1371	AUROC: 0.940
Lammers et al. [49]	2022	Predicting risk of requiring massive Transfusion	RF	HR, RR, DBP, SBP, SpO_2_, Temperature, INR, Hematocrit, Platelet, pH, mechanism of injury, GCS, AIS, ISS	DoDTR	22,158	AUROC: 0.984
Chen et al. [50]	2008	Determining hypovolemia in patients	Linear ensemble classifiers	HR, RR, DBP, SBP, SpO_2_	Data from hospital/ER records	898	Accuracy: 0.760
Convertino et al. [51]	2011	Determining patients at greatest risk of ongoing hemorrhagic shock	undefined ML algorithm	SBP, DBP, RR, blood pH, base deficit	Data from subjects under LBNP	190	Accuracy: 0.965
Rickards et al. [52]	2015	Determining hypovolemia in patients	undefined ML algorithm	HR, stroke volume, ECG, heat flux, skin temperature	Data from subjects through various exercises under LBNP	24	Accuracy: 0.926
Davis et al. [53]	2022	Intracranial hemorrhage detection	NLP tool	CT scan images	Data from hospital/ER records	200 scans (25,658 images)	Precision: 0.730
Ginat et al. [54, 55]	2020, 2021	Intracranial hemorrhage detection	NN software	CT scan images	Data from hospital/ER records	8723 scans	Accuracy: 0.965
Davuluri et al. [56]	2012	Hemorrhage detection and image segmentation model	SVM	CT scan images	Data collected from hospital/ER records	12 scans (515 images)	Accuracy: 0.943
Perkins et al. [57]	2021	Prediction tool for detecting TIC	BN	HR, SBP, temperature, hemothorax, FAST scan, GCS, lactate, pH, mechanism of injury, fracture assessment	Data from hospital/ER records	1091	AUROC: 0.930
Li et al. [58]	2020	Prediction model for acute traumatic coagulopathy	RF	RBC count, SI, base excess, lactate, DBP, pH	Emergency Rescue Database	1014	AUROC: 0.830

*AIS* abbreviated injury scale, *ANN* artificial neural network, *BMI* body mass index, *BN* bayesian network, *DBP* diastolic blood pressure, *DNN* deep neural network, *DT* decision tree, *ECG* electrocardiography signal, *FAST* Focused Assessment with Sonography for Trauma, *GCS* Glasgow Coma Score, *HR* heart rate, *INR* international normalized ratio, *ISS* injury severity score, *LASSO* least absolute shrinkage and selection operator, *LBNP* low body negative pressure, *MAP* mean arterial pressure, *MLP* multi-layer perceptron, *NLP* natural language processing, *PR* pulse rate, *PT* prothrombin time, *PTT* partial thromboplastin time, *RBC* red blood cell, *RF* random forest, *RR* respiratory rate, *SBP* systolic blood pressure, *SCS* Simplified Consciousness Score, *SI* shock index, *SpO*_*2*_ oxygen saturation, *SVM* support vector machine, *TOP* trauma outcome predictor, *WBC* white blood cell

Further analysis reflects an overwhelming portion of retrospective studies (*n* = 72), which utilized data from various hospital and trauma databases to develop and train ML algorithms. With the use of structured data (patient demographic, physiological and laboratory data, injury/trauma scores, and other information relating to the trauma), the models can be trained, tested and validated accordingly. Unstructured data used in the papers is comprised of neuroimaging data pertaining to Computed Tomography (CT) and Focused Assessment with Sonography for Trauma (FAST) scans for hemorrhage detection, or to assess the trauma severity [53, 56, 57, 59, 60]. The studies are summarized in detail under each category below.

The majority of the studies that fell in the outcome prediction category were designed to predict in-hospital mortality, survival, and/or comorbidities due to the hemorrhagic trauma. Five study designs predicted mortality at specified time points after patient admission such as within 24 h, 7 d, 1 month or 1 year [27–29, 61, 62]. All included studies reported increased discrimination when using ML models to determine those patients who survived from those that did not. Alternatively, the main focus of the studies categorized under risk assessment and injury severity was to develop and assess the severity of a person’s injury and assess their need of care over other patients. Studies on transfusion focused on predicting the need for transfusion/MT (*n* = 9), while a few predicted specific needs for resuscitation on arrival. Detection of hemorrhage in trauma patients were conducted either using clinical variables or imaging scans. Moreover, multiple studies also investigated the possibility of detecting hemorrhages in patients using imaging data [FAST, non-contrast CT (NCCT), CT scans]. These studies can be divided into those that investigated and developed algorithms for intracranial hemorrhage detection and those for hemorrhage detection in pelvic trauma patients.

### ML model development and performance metrics

With consideration of the various study groupings, the models developed in the included studies were network, regression, tree, and kernel-based (Table 2). Based on the literature search, regression-based models were used most frequently (*n* = 32), followed by network-based (*n* = 31), tree-based (*n* = 29), and kernel-based (*n* = 14). Additionally, logistic regression (LR) models were most commonly used, either as a comparison or for use as a feature reduction method, such as penalized LR models (least absolute shrinkage and selection operator, Ridge, and ElasticNet regression), Cox regression, Poisson regression, and Stepwise regression. Studies that used network-based models, mainly implemented a variety of feed-forward neural network (NN) methods such as artificial neural network (ANN), deep neural network (DNN), and multi-layer perceptron (a subset of DNNs). Finally, for tree-based models, various random forest (RF) and decision tree (DT) methods were implemented.

**Table 2:** Different algorithms used in trauma study topics

Study topic	Category	Method in search	References
Different outcomes in trauma	Regression	LR	[34, 37, 41, 62–72]
	Network	DNN, ANN, MLP, RBFN, Predictive Hierarchical Network, Polynomial NN, RSNNS	[27, 36, 37, 41, 63–68, 70, 71, 73–80]
	Tree	CART, DT, RF, Recursive Partitioning Algorithm, OCT, Bayesian DT, unpruned C4.5 tree (J48), Archetypal DT	[27, 29, 30, 37, 41, 63, 67, 68, 70, 72, 81–85]
	Kernel	SVM, SMO, Polynomial Kernel, SVM Radial	[29, 37, 63, 66, 71, 72, 81]
	Ensemble	SuperLearner	[86]
	Boosting	XGBoost, Gradient Boost	[32–35]
	Other	LDA, ER, FIS, Inference methodology	[27, 28, 63, 66, 72, 81, 87]
	Bayesian	GNB, NB, BBN	[27, 37, 69, 72, 82, 84, 88, 89]
	Unmentioned/commercial ML algorithm, novel scoring systems	Deep-FLAIM, UKTRISS, TOP, 4TDS, EDI	[27, 31, 36, 61, 90, 91]
	Classification	KNN, Maximum a Posteriori	[27, 37, 72, 84]
Risk assessment	Regression	LR, MLR	[39, 40, 42, 92–96]
	Network	ANN, MLP, DNN, Dirichlet DNN	[38, 40, 42, 44, 93, 97, 98]
	Tree	RF, DT, Boosted Tree	[40, 42, 43, 92, 95, 96, 98, 99]
	Kernel	SVM, SVMR	[39, 95, 97, 99]
	Bayesian	BBN, NB	[96, 98]
	Boosting	XGBoost, Adaboost	[94, 97, 98, 100]
	Ensemble	Bagging	[97]
	Other	Generalized Linear Model, LDA	[99, 101]
	Classification	KNN	[97]
	Unmentioned/commercial ML algorithm, novel scoring systems	CRI, MGAP	[45, 102]
Transfusion	Network	NN	[49, 60, 103]
	Kernel	SVM	[49]
	Boosting	XGBoost	[48, 49]
	Tree	Classification and regression tree, Recursive partitioning analysis	[48, 49, 104, 105]
	Regression	Logistic regression	[48, 49, 106]
	Unmentioned ML algorithm, commercial ML software, novel scoring systems	CRI, MASH, BRI	[47, 52, 107, 108]
Hemorrhage detection	Network	Multi-scale attentional network	[59]
	Ensemble	Ensemble classifier	[109]
	Regression	Poisson regression	[110]
	Kernel	SVM	[56]
	Unmentioned/commercial ML algorithm, novel scoring systems	BRI	[111]
	Other	Linear/non-linear density model, NLP Linear Classifier	[51, 53, 112]
Coagulopathy	Regression	LR	[113]
	Tree	DT, RF	[58, 113, 114]
	Bayesian	BN	[57]
	Unmentioned/commercial ML algorithm, novel scoring systems	Caprini RAM	[113]

*ANN* artificial neural network, *BBN* bayesian belief network, *BN* bayesian network, *BRI* bleeding risk index, *CART* classification and regression tree, *CRI* critical reserve index, *DNN* deep neural network, *DT* decision tree, *ER* evidential reasoning, *FIS* fuzzy inference system, *LDA* linear discriminant analysis, *LR* logistic regression, *MASH* military acute severe haemorrhage, *MGAP* Mechanism, Glasgow coma scale, Age, and Arterial Pressure, *MLP* multi-layer perceptron, *MLR* multi-linear regression, *NB* Naïve bayes, *NLP* natural language processing, *NN* neural network, *OCT* octree method, *RAM* risk assessment model, *RBFN* radial basis function network, *RF* random forest, *RSNNS* stuttgart neural network simulator, *SMO* sequential minimal optimization, *SVM* support vector machines, *TOP* trauma outcome predictor, *TSM* trauma severity model, *UKTRISS* United Kingdom Trauma and Injury Severity Score

Studies from the five categories showcased similar model selection for their respective outcomes. The studies aimed at trauma outcome mostly used a network-based algorithm, specifically DNN, for predicting the outcome of a patient following a traumatic incident. Alternatively, risk assessment, transfusion and coagulopathy prediction all found tree-based models with common usage. Due to the lack of included studies for hemorrhage detection, prediction of transfusion and coagulopathy, a discernable common model cannot be directly stated.

In general, a similar recipe was used for model development. This process involved collecting either retrospective data through a database/hospital record, or prospectively through a trial, after which the features were selected through various optimization methods. For majority of the cases, the data were split into a training and testing set for cross-validation, and hyper-parameter tuning was conducted to find the best performing model, and its performance metrics were calculated. Overall, all of the models provided a significant improvement in the goal of their study, by either developing a model that outperformed a scoring standard or another previous model, or provided an efficient decision-making tool in quick-assessment cases such as triaging or forecasting the need for specific interventions to ensure patient survival.

A large population of the model development studies (*n* = 66) conducted validation of ML models. Resampling methods, such as holdout methods (testing-training split) and k-fold cross validation were the most frequently used. Eighteen studies did not provide any information on any validation performed on the model. Twelve studies utilized a secondary cohort from a different database as a testing set for the models [45, 57, 58, 61, 68, 73, 88, 90, 93, 96, 110, 115]. Finally, out of the included studies, four studies performed an external validation on a previously developed ML model [31, 47, 61, 115].

To evaluate the performance of the developed model, various metrics were utilized across the studies. The most common metric was the AUROC curve followed by accuracy, sensitivity, and specificity. Model performance metrics varied depending on the outcome being predicted, ML method used and the prediction window. Some studies developed additional models and/or used trauma/injury scoring standards to compare and evaluate the performance of the developed algorithm [27, 28, 30, 34, 36, 37, 41, 61, 65, 67, 71, 73, 75–77, 80, 83, 85, 88–90, 93, 108, 113].

### Dataset collection

Data for developing the ML algorithms were collected via three main methods: (1) trauma databases, (2) Hospital record and (3) prospectively in a lab/simulation setting. Fifty of the included studies used de-identified trauma patient data from various local and globally available databases. The most common database was the National Trauma Data Bank (NTDB), the largest aggregation of U.S. trauma data. Other databases such as the Trauma Audit and Research Network, American College of Surgeons Trauma Quality Improvement Program were similarly utilized for data collection in model training. This data was then filtered using inclusion and exclusion criteria, and the features were selected based on the purpose of the study. For example, Tsiklidis et al. [35] obtained data from the NTDB to develop an ML classifier for predicting survival probabilities. Demographic data (age, gender, alcohol use, and comorbidities) and physiological data [heart rate (HR), respiratory rate (RR), systolic blood pressure (SBP), and diastolic blood pressure (DBP), etc.] were extracted from the database and missing data or improper data were excluded. Permutation importance method was used for evaluating which features were significant for predicting the outcome, and reduced the features used from 32 to 8.

Furthermore, 35 studies also utilized data from regional/local hospitals. As a result, there may be fewer patient data available for development, and in most cases, these studies excluded any dataset with missing variables, which consequently reduced the sample size. Finally, four prospective studies used lab data from selected subjects. For example, Rickards et al. [52] conducted a study on the use ML algorithms to track changes in Shock Volume, through progressive low body negative pressure and exercise. Twenty-four volunteer subjects who were normotensive, nonsmoking, and not pregnant were selected for the study. A major drawback with prospective studies in such cases is the low population set, resulting in a model lacking in variance, especially if the data is unbalanced. Furthermore, it also prevents a proper testing set, which makes it more prone to over-fitting to the training dataset.

Study populations in each of the included studies varied significantly. The lowest retrospectively used population set was 70 subjects by Chapman et al. [114] who collected rapid-thromboelastography tracings from blood samples of end-stage renal disease patients (*n* = 54) and trauma patients requiring a MT (*n* = 16) between May 2012 through April 2013. The highest population sample using 2007–2014 NTDB data was 2,007,485 in a retrospective study by Cardosi et al. [32, 114]. In the prospective studies, these values were even lower, with a sample size of 24 in the aforementioned prospective study by Rickards et al. [52] collected through human trials. In total, 30 studies used a population under 1000, 26 studied had a population between 1001 and 10,000 and 31 studies used a population over 10,000 patients [52].

### Feature frequency

**Fig. 2:**
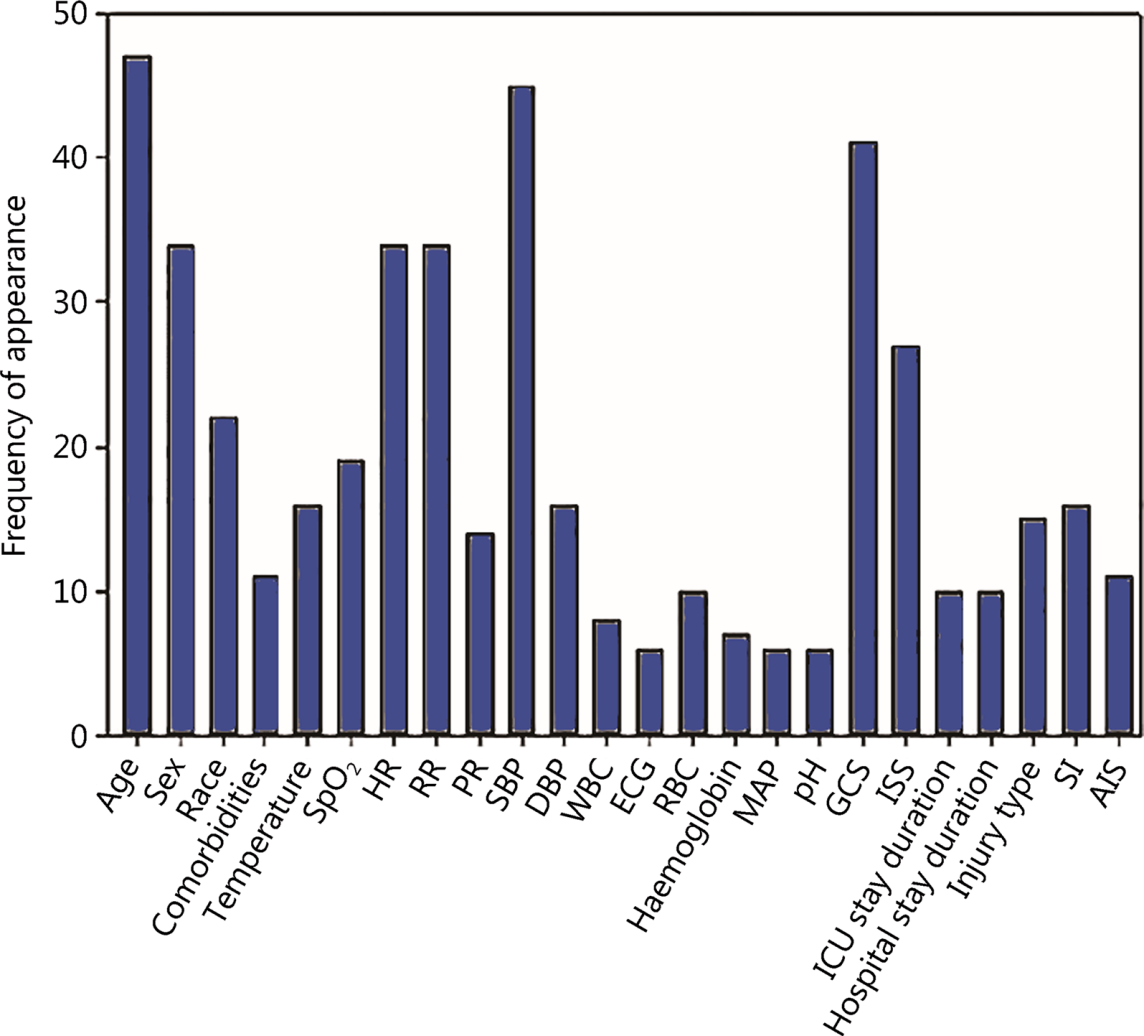
Frequency of features in included studies. HR heart rate, RR respiratory rate, SpO_2_ saturation of oxygen, SBP systolic blood pressure, DBP diastolic blood pressure, ECG, electrocardiography, RBC red blood cells, MAP mean arterial pressure, GCS Glasgow Coma Score, ISS injury severity score, SI shock index, AIS abbreviated injury scale

Several commonly collected variables for ML training were identified, and could be divided into demographic, physiological, and additional data **(**Fig. 2). Common demographic data included age, sex, ethnicity, hospital/Intensive Care Unit (ICU) stay duration and whether the patient suffered any comorbidities (e.g., alcohol use, smoking, any cardiovascular diseases, any hereditary diseases, any current conditions). Physiological variables include physiological or laboratory data such as HR, SBP, DBP, RR, temperature, blood volume, electrocardiography, oxygen saturation (SpO_2_). Finally, other relevant variables pertaining to the outcome of the study such as the injury location, type of injury, and common injury assessment scoring systems [e.g., Glasgow Coma Score (GCS) and shock index], units of red blood cells (RBC) and white blood cells, fresh frozen plasma (FFP), and platelets were also included.

Based on complete analysis, age, systolic blood pressure, GCS, sex, HR, RR, SpO_2_, temperature, injury severity score, shock index, and type of injury were the most common features presented in the papers. Variables such as fresh frozen plasma, hematocrit, thromboplastin and photoplethysmography were not commonly used in the limited number of studies on coagulopathy and transfusion prediction. Studies conducted by Ahmed et al. [27] and Kuo et al. [29] utilize additional physiological data and laboratory data such as white blood cells count, packs of RBC given, FFP as variables for trauma outcome prediction, which increased their inclusion frequency [27, 29].

Among the included studies, 47 studies reported missing values in their datasets, out of which 24 excluded these data. In terms of imputation methods, mean imputation was the most used among the 33 studies which mention how the missing values were handled. Other imputation methods used were iterative or multiple imputation, ElasticNet regression, optimal imputation, chained equation imputation, and median imputation [30, 35, 44, 62, 70, 71, 80, 94, 97, 110, 113]. For dealing with imbalanced data, 6 studies addressed it with the most commonly used method being Synthetic Minority Over-Sampling Technique [49, 63, 72, 81, 91, 99].

### Feature selection of predictor variables

Multiple studies investigated how different numbers, types and sets of features affected the ML model’s performance. Almost all studies concluded that increasing the number of features did not necessarily improve performance. Several feature selection methods were identified, such as penalized logistic regression (least absolute shrinkage and selection operator, Ridge, and ElasticNet), Cox regression, χ-square, permutation importance. For example, Tsiklidis et al. [35] used the permutation importance method to select features that would be most selective of outcome and reduced the number of features from 32 to 8 easily measurable features.

Given the time sensitive nature of the study outcomes of trauma, variables that are easier to measure during transit to the evidential reasoning (ER, pre-hospital setting) or upon admission are fundamental for use as predictor variables. While laboratory or clinically acquired variable data adds value that improves performance, the delay in acquiring this information could hinder potentially life-saving intervention.

Easily accessible variables such as age, sex, race, HR, RR, SBP, as well as GCS, injury severity score (ISS), and the type of injury were also more commonly used for model development. Vital signs during transport and/or during ER admission reflect a higher relevance for outcome studies. Liu et al. [39] showed the significance of vital sign measurements, and heart rate complexities to predict whether life-saving intervention was required, and saw that continuous measurement of vital signs allowed for sensitive prediction of life-saving intervention outcome. Kim et al. [41] proved that Simplified Consciousness Score was the most important feature for survival prediction in the LR, RF and DNN models. Kilic et al. [28] and Pearl et al. [78] found that physiological variables from the scene had little to no impact on the performance of their model; Kilic also noted that response to resuscitation had an important effect on trauma mortality [28, 78]. Paydar et al. [97] reported that DBP was more important than SBP as a predictor for mortality, while Walczak et al. [103] found that SBP was the second most contributing variable for transfusion prediction.

### Comparisons with injury scoring standards

Trauma and injury scoring systems can be crucial for injury characterization, especially in terms of assessing and providing prognosis for trauma incidents [116]. While current scoring systems are substandard, triaging departments often utilize them to evaluate patients efficiently by separating them on the degree of injury and threat of mortality and/or morbidity [117]. This presents an inherent standard for the measurement of trauma and/or injury as well as for making accurate prognoses. Trauma scoring systems can be divided by the type of data used to assess injury and trauma such as physiological- and anatomical indices, and combined systems that use combined anatomical and physiological data [17].

Performance analysis of models reported in the literature elucidates how novel ML algorithms outperform current injury scoring mechanisms, as well as improving the overall prediction for need of ICU care/outcome. For example, ISS accounts for anatomical lesions without the consideration of vital signs. Moreover, it cannot be computed on scene and is viewed as an ineffective predictor for ICU care. A DT model was developed by Follin et al. [45] aimed to diminish that problem by utilizing vital signs-based variables, resulting in a highly sensitive model with good performance. Trauma and Injury Severity Score (TRISS) is a combined scoring system that incorporates ISS, Revised Trauma Score, and age, and is a universal tool to predict the outcome of a trauma patient. Several studies established models with greater predictive performances than TRISS, demonstrating a shift towards the creation of an improved outcome prediction model [27, 28, 30, 37, 65, 67, 71, 73, 76, 77, 80, 83, 85, 89].

Utilization of these scoring systems as predictor variables offers a paradoxical approach to model prediction. As mentioned previously, some of these systems cannot be computed on scene, and require the patient to arrive at the emergency room before providing the class and score of the trauma and injury. Since the purpose of these models is to be used as an initial management and rapid decision-making system, using these scores not only creates a manual element (as a health care provider needs to classify the injury subjectively), but also delays the time it takes to receive an output from the model. It is more beneficial to use these for comparative purposes (e.g., comparing the predictive accuracy of a triage classification model against the hospital triaging using ISS), as it can better showcase the model’s efficiency and accuracy in tandem to the scoring system. However, the impact of time to treatment versus time spent on-scene on a patient’s outcome continues to be a matter of contention, especially with regard to the notion of a “golden-hour” of care. Small sample sizes, inconsideration of injury severity and/or treatment given during pre-hospital transit could be used to argue the ineffectiveness of pre-hospital care on the patient’s outcome [118–120]. Large, well-controlled studies could provide insight into the impact of these timings on patient care, or to develop mortality prediction models using these time measurements as features.

Numerous studies lacked evaluation of the model using external test datasets and reported performance only through the test set split from the original dataset. Validation is a critical step in optimizing a model with elevated robustness for predictive tasks with data from a wider population. Specific studies utilized the retrospective data and separated a second cohort as a form of external testing, either using data from a different time range or a different database [45, 57, 58, 68, 73, 88, 90, 96, 110]. One study conducted a retrospective study using trauma patients between January 1, 2012, and December 31, 2014, while creating a second cohort using patient data between January 1, 2015 and August 1, 2015 [45]. The performance of the DT model decreased between the original cohorts with the testing cohort (0.82 vs. 0.79). Another study reported a worse performance between two identified cohorts (training/testing cohort used the NTDB and Nationwide Readmission Database for external validation), changing significantly from 0.965 to 0.656 [90]. This could be remedied by calibrating the model according to the Nationwide Readmission Database but highlights the increasingly complex and non-linear nature of emergency modeling. Furthermore, several studies tended to exclude patient data with missing fields, which introduces a lot of error through bias. Countermeasures such as utilizing various imputation methods could aid in providing a greater range of data. Further external testing and cross-validation, especially one conducted in a long prospective study should be conducted on these models to further develop and optimize them.

### Comparisons of ML models with different studies

The studies from each of the categories provide value for each respective application. For trauma outcome and risk assessment, the models aim to provide a scoring metric to identify a patient’s probability of survival given their injuries, as well as aid in providing quicker an automatic sorting methodology of patients needing rapid treatment. Studies focusing on models for transfusion aim to develop automatic identification of patients in need of transfusion, as well as the specific requirements for the patient. Based on the clinical and lab data, similar automatic prediction of trauma induced coagulopathy and detection of hemorrhage have been conducted. The greatest value that these studies would provide in the field of medicine is the absence of human intervention and prediction to provide individual care, which is extremely valuable in remote or inaccessible locations.

Model comparisons are made broadly through AUROC; however, it is imperative not to make direct comparisons between two models due to the extensive variability of the models made from different studies, different data types and sets, different algorithms, and most importantly, different predictive outcomes. Therefore, the comparisons being made are considering how well the model was able to perform the specific task it was assigned, and this is what is being compared. Table 3 outlines the general statistics of the included studies in their respective categories, as well as a model that was able to best perform the specific task based on the study design.

**Table 3:** General statistics of the studies included in the review

Study topic	Number of papers	Range of year	Range of patients/data used	Best performing models (AUROC)	Mean performance ± SD
Different outcomes in trauma	45	1995–2022	32–1,511,063	Performance of max EDI after 24 h for mortality (0.98) [61]	0.91 ± 0.06 (*n* = 36)
Risk assessment	18	2009–2021	73–2,007,485	Performance of RF model trained using ISS, AIS chest, and cryoprecipitate given within first 24 h (0.97) [92]	0.88 ± 0.07 (*n* = 13)
Transfusion	11	2015–2021	477–12,624	Performance of RF model using age, gender, mechanism of injury, involvement in explosion, vital signs (0.98) [49]	0.87 ± 0.09 (*n* = 8)
Hemorrhage detection	11	2007–2021	24–368,810	Performance of Poisson Regression model using epidemiological data, GCS, SBP, DBP, HR, haemoglobin, amount of RBC packs, platelets and fresh frozen plasma transfused, transfusion timing, and coagulation tests results (0.92) [110]	0.92 ± 0.07 (*n* = 6)
Coagulopathy	4	2014–2019	54–18,811	Performance of BN model using HR, SBP, Temperature, Hemothorax, FAST result, GCS, Lactate, Base deficit, pH, mechanism of injury, pelvic fracture, long bone fracture (0.96) [57]	0.89 ± 0.08 (*n* = 3)

*AIS* abbreviated injury scale, *DBP* diastolic blood pressure, *EDI* epic deterioration index, *FAST* focused assessment with sonography for trauma, *GCS* Glasgow Coma scale, *HR* heart rate, ISS injury severity score, *RBC* red blood cell, *RF* random forest, *SBP* systolic blood pressure

Various triaging models developed were included in our review, out of which RF models were more commonly used. The best performing models were reported by Pennell et al. [99], where RF, support vector machines (SVM), and Generalized linear model (GLM) produced AUROC values up to 0.99 in both low- and high-risk cases (GLM produced an AUROC of 0.96 for both cases). Similarly, Paydar et al. [97] reported their Bagging and SVM models having high AUROC values of 0.9967 and 0.9924 respectively, using GCS, Backward Elimination, and DBP as predictive variables. Studies reviewed that developed testing sets from external databases reported a decrease in their model performance. This is evident in the models developed by Larsson et al. [100], where the models showed a decreased predictive performance after using an external testing cohort. The study highlighted the XGBoost model decreased in performance when using the cohort from the NTDB dataset (AUROC of XGBoost was 0.725 using the SweTrau vs. 0.611 using NTDB, while the LR model was 0.725 and 0.614).

For those studies focused on transfusion, tree-based modes and more specifically DT models were the most common. Majority of the studies discussed the need for transfusion/MT after trauma. Of this, the RF model by Lammers et al. [49] exhibited optimal performance (AUROC value of 0.984); other high performing models included SVM and LR model yielding an AUROC value of 0.9677 and 0.9637 respectively. Similar LR models by McLennan et al. [106], Mina et al. [46], and Feng et al. [48] also yielded high AUROC values (0.93, 0.96, and 0.80 respectively) [46, 48, 106]. However, validation on the model by Mina et al. [46] showed a substandard performance (AUROC value of 0.694), suggesting that the other models might be over-fitted to their respective training datasets and not generalizable. Additionally, this could also suggest that these models are not generalizable since they lack time-dimensionality as a factor. For example, a model trained using vital sign data of patients over a specified time interval could result in higher sensitivity. The large amount of LR models that produced high performance highlight a preference and strength of regression models for their discriminative potential using simple, readily accessible data.

Walczak was the only study that investigated the prediction of transfusion needs of various transfusion products [103]. The ANN models exhibited high accuracy, sensitivity and specificity for each blood product (RBC, FFP and platelets models had an accuracy of 0.6778, 0.8264, 0.705 respectively). Over prediction of blood products was the most commonly observed error in the ANN model. Future studies on hemorrhagic trauma and transfusions should further investigate specific blood product predictions. Blood product prediction models like these could be very effective for remote field sites, allowing trauma physicians to cache away specific types of blood supply based on their frequency of use. Furthermore, well-developed and validated models could aid in life-threatening situations by allowing hospital sites to prepare specific amounts of blood products before patient arrival.

Out of the studies that predicted any case of bleeding in trauma patients, Lang et al. [110] had the best performing models for detection of hemorrhagic shock and traumatic brain injury yielding AUROC values of 0.92 and 0.97 respectively. Linear models (especially regression models) generated the best performing models for these studies. Chen et al. [50] found that using HR, SBP, SpO_2_ as predictor variable yielded the best AUROC value of 0.76. Alternatively, Chen et al. [112] delivered the same performance using SI as a predictor variable; they also found that HR, SaO_2_, and DBP to be the best multivariate discriminator between major hemorrhaging and any control cases. Moreover, the model’s performance slightly decreased when using a dataset containing missing values (AUROC of 0.76 vs. 0.70). This study showcases the benefit of using a linear ensemble classifier being their robustness in handling missing values, compared with other models. Considering the mentioned studies found linear classifier models yielding high performance, creating a combination of classifiers into a linear ensemble model could offer a robust, high functioning decision-support tool.

Based on the included studies that focused on the field of coagulopathy, tree-based models were the most common. The BN model developed by Perkins et al. [88] outmatched the other models, especially when considering that the externally validated model yielded an AUROC value of 0.93, compared with an AUROC of 0.830 and 0.800 from the RF models assembled by Li et al. [58] and He et al. [113] respectively. Given the lack of included studies focused on ML for prediction of trauma-induced coagulopathy (*n* = 4), no conclusive statement can be made about the best model for trauma related coagulopathy. The included studies showed a general imbalance in the kinds of research being conducted. Research into detection and automated assessment of coagulopathy has not been investigated in detail, which prevents meaningful cross-comparison discussions. Moreover, it also prevents any meaningful conclusions to be made on the best features, or the best model for these specific topics.

### Strengths and weaknesses of models

The application of ML models in hemorrhagic trauma shows potential for use in medical and clinical routines due to their established high predictive and decision support performances. Included studies utilized various types of regression, trees, network, and ensemble models, which can be used to identify certain strengths and drawbacks of using these specific algorithms. Kim et al. [41] found that the RF and NN have more discriminative power due to the nonlinear relationship between the input and output parameters. The combination of their discrimination power, along with the nonlinear characteristic of the NN shows an improved performance compared to the LR models. Chesney et al. [64] also found that the ANN offered a higher predictive accuracy as well as a higher sensitivity compared to the LR models which were much better at outcome discrimination. Kong et al. [66] and Lammers et al. [49] found that LR models can identify the predictor variables that show a higher statistically significant correlation with a particular outcome, and presents an easy-to-interpret modeling method. Furthermore, Scerbo et al. [43] found that LR was not able to adapt or control to allow for slight leniencies; in the case of their study, the LR did not attempt to over-triage to error on the side of caution.

Chen et al. [50] stated that the ensemble classifier performed better than a single linear classifier, especially when applied through multiple testing/training trials. These ensemble classifier offers statistical, computational, and representational advantages compared to a single classifier, which means that it would have a more consistent performance throughout a broader population. Similarly, Roveda et al. [70] suggested that other ensemble algorithms would provide even better results than their RF model (also an ensemble model). Seheult et al. [105] recommend the use of ensemble ML methods, due to their decreased risk of over-fitting (leading to the models with a low variance but high bias), unlike DT models which have a high over-fitting (i.e. models with high variance but low bias) potential due to a high dependence on the training set. For DT models, Feng et al. [48] found that the inclusion of more parameters resulted in a DT model with higher predictive performance.

Some studies implemented various models/techniques for performing their proposed task and compared the performance among these models. For example, Ahmed et al. [27] created a mortality prediction ML model using several clinical and laboratory-based variables. The proposed DNN “FLAIM” model was compared with other models like Linear Discriminant Analysis, Gaussian Naïve Bayes Classifier (GNB), Decision Tree (DT), k-nearest neighbor (KNN), as well as other trauma and injury scoring standards. They found that the DNN-FLAIM model outperformed all the other ML models, with an AUROC of 0.912 compared to the TRISS of 0.903, and GNB of 0.836. Similarly, Sefrioui et al. [37] evaluated various models for predicting patient survival using easily measurable variables. RF, KNN, C4DTs (J48), LR, Naïve Bayes (NB), ANN, SVM, and Partial Decision Tree models were used, and the RF model showcased the highest AUROC, accuracy, and specificity, while the SVM model yielded the highest sensitivity. Furthermore, the SVM model reported by Sefrioui et al. [37] yielded an AUROC and accuracy of 0.931 and 0.969, respectively.

While there may be certain advantages and disadvantages of choosing one model over another in specific applications, these models are often limited by performance by the information and data points that were used to train them. The ability of a model to provide accurate personal predictive monitoring (PPM) is largely dependent on developing an algorithm that can provide a superior AUROC value (resultant of higher specificity and sensitivity). As such, this gives rise to deceptively high-performance metrics, since the majority of the algorithms are developed for the identification of clinical events using retrospective data.

### Limitations presented by the included studies

Most studies have been conducted using retrospective data. In contrast, prospective studies often utilize a low sample population that is accrued over a long-time span. A possible workaround may be to train models on retrospective datasets and then be tuned with different retrospective and prospective datasets to create a more robust, generalizable model. However, this generalization fails at identifying unique and underlying physiological conditions that may not be evident through vital sign/laboratory data.

The data and the model, the time feature, and the personalized predictive monitoring are three ideas that go together for developing AI systems for medical care and should be conceptualized if one is to develop AI systems for trauma and medical care. One option to implement them could be to put them into a real-time monitoring system to generate a personalized temporal predictive system. To implement the time feature in ML solutions, one can train a temporal model with real-time data to generate a temporal prediction model. However, end-point data could also be translated to develop a temporal solution (which is significant considering that majority of the included studies utilized end-point data). Indeed, the use of real-time data is already evident in several studies that aim to utilize non-invasive techniques in measuring pulse arterial waveform to develop a real-time tracking solution [51, 52, 121, 122]. Work by Convertino et al. [123] highlights the sensitive nature and monitoring approach that arterial waveform feature analysis may provide for earlier and individualized assessment of blood loss and resuscitation in trauma patients. Future studies could develop such models in an effort to compare the performance of utilizing temporal and end-point data, or to further develop a reliable real-time PPM. Furthermore, retrospective data being used leads to a major limitation of being largely dependent on the data that is being used. Due to the retrospective nature and the varying data that is provided by the studies, these performance metrics may greatly vary from dataset to dataset. As such, the concepts of ‘time’ and dataset quality hold the greatest weight on overall model performance, and as such are elements that should be classified or standardized for model development. Finally, the majority of aforementioned ML models are based on population averages obtained from large subject pools that mask inter-patient variability. Addressing inter-patient variability is the objective of personalized medicine [124]. Future research should aim to develop increased model explainability to allow for each sample to be analyzed, in order to identify which feature has a significant impact on the predictive output. This prompts readers to wonder the kind of data to be collected (to provide the most accurate prediction for initial admission) and its impact on PPM, and we hope future authors investigate this concept in detail.

Demonstration of high performance and accuracy metrics in large subject population prospective randomized controlled trials (RCTs) would be the best way to direct development towards the use of these models as medical standards. Future studies should investigate the use of their models on prospective datasets, as it would only further helps with validation. Comprehensive clinical datasets are often difficult to obtain even with the rapid increase in available data, as it is limited by specific patient testing and recording. Few studies randomly generated injury data and made appropriate injury assessment and labelling to augment the overall dataset [98]. Augmentation allows for a larger and more randomized albeit synthetic dataset, which would ultimately improve model performance. Class imbalance is another frequent obstacle in available datasets, with the majority outcome being a predominant class of the outcome predictor. Studies often utilize oversampling techniques such as Synthetic Minority Over-Sampling Technique in generating samples for the minority class [63, 81]. Other studies targeted specific inclusion and exclusion criterions to include data from specific trauma population with variables focusing on their aim of research, while a few studies involved an unclear population set. This was evident in the studies that focused on hemorrhage detection and prediction, where traumatic brain injury (TBI) as a subgroup of trauma related injuries was disclosed, hence the data from patients with non-TBI or intracranial hemorrhage patient data were used. These datasets could include blunt trauma patients, in-patients or patients with complications following a different pathology. In the case of the study by Ginat et al. [54], all urgent NCCT scans were used in the training and testing of the ANN model [54]. Among the true positive scans were patient initial scans, follow-up cases, trauma/emergency cases, inpatient, and outpatient cases. Although these cases accounted for 70.7% of the dataset, the accuracy for all cases used was lower than that of trauma/emergency cases only (0.934 and 0.961 respectively). There are guidelines listed in literature regarding the type and amount of input data required for each type of ML model [125]. They report that regression would require 100–1000 data points, while regularized regressions, SVM, DT, RF, and KNN models require 100–1,000,000 data points. In contrast, NN models require greater than 10,000 data point amounts. Aiming for a larger data population and for model development can lead to lower estimation variance, and consequently a better predictive performance. Due to the simplified nature of regression-based models, only a limited amount of input variables can be used to predict an outcome, while NN models would require a much greater feature set. Twenty of the studies (22.5%) included in this review used < 500 study participants, and the training with such low dataset could lead to an over fitted model, increasing the prediction error. Gathering additional retrospective cohorts, performing data augmentation methods on the datasets, utilizing parameter regularization, or implementing ensemble models are recommended in improving overfitting and final accuracy of the system. Additionally, data that incorporates time as a factor/variable would greatly aid in improving the overall sensitivity and specificity of the model. Performance metrics used in different studies varied from including sufficient metrics to characterize the ML models, to some not including any. This variance in metrics, as well as an absence of standard reporting metrics for these models prevents any meaningful comparisons from being made for all the studies. The vast majority of the papers report the AUROC of the model, indicating an unspoken standard metric that is emerging in literature. Metrics such as F-measure and precision (positive predictive value) were less commonly reported in all the included studies, given that many studies focused on a multiclass classification prediction driven model. F-score is beneficial as it yields a better estimate of the model’s accuracy, through the calculation of the harmonic mean of the precision and sensitivity (or recall). While sensitivity was a commonly reported metric amongst the included studies, incorporation of the F-score (and by association, precision) in future studies would prove to be useful in providing a better measure of a model’s performance.

### Limitations of this review

There are several limitations of this review. Firstly, this study merely includes publications written in English, which may have caused publication bias. Secondly, this review focuses on the literature for hemorrhagic trauma, and would be excluding papers that while may provide rigorous models, falls out of the scope of this review. Thirdly, the included studies may have biases themselves, which may have caused bias in results. Publication bias and selective outcome reporting could influence the results of this review, as all the included studies reported high performing models, albeit some with inferior performances to common scoring methods. Furthermore, this review does not consider many of the intricate and nuanced ML concepts that might be beneficial for analyzing and comparing the studies included. Some of these concepts, such as uncertainty and explainability of these models, would provide more context to the sensitivity of the model in performing on other dataset and/or the ability of the models to be conceptualized and understood by front staff providers. These concepts may be discussed in future reviews, as this review aimed to provide an overarching survey on the current studies surrounding the topic. As mentioned in this review, few studies offered comprehensive model performance metrics, which resulted in undiscernible performance comparisons throughout the study. Moreover, the lack of external testing sets and generalizability of the models would result in inflated performances of some models, which would result in this study incorrectly reporting the highest reporting models. Finally, the lack of studies investigating prediction of transfusion, hemorrhage, and coagulopathy prevent any meaningful comparison and conclusion to be made regarding models used in those studies. Future research into the comparison and application of ML algorithms using different datasets in RCTs would further support the implementation of ML technologies for trauma care.

## Conclusions and future directions

This review demonstrates that ML models have capabilities that enable more accurately predicting situations concerning traumatic hemorrhage than currently used systems. Use of small variable sets that are easily accessible has become a standard for producing high performing and accurate models in trauma. Although many of the included models outperform traditional scoring systems, the evaluation of their performance is limited by a conforming population and a retrospective dataset. While these models have the potential to provide clinical decision support, there is a need for standardized outcome measures to allow for rigorous evaluation of performance across models, as well as to address the intricacies concerning inter-patient variability. Further consideration on the impact of the features on the predictive output, as well as feature/model explainability are crucial for developing rapid personalized trauma diagnosis and treatment models. Identifying key features and/or attributes to specific regions of trauma care could be crucial in developing a rigorous model capable of providing personalized predictive monitoring through precision-based medicine (PBM). Indeed, emerging studies have already introduced the implementation of ML within the context of goal-directed and personalized care [124, 126, 127]. Future research would need to investigate feature significance on model accuracy, as well as the implementation of these models into clinical routine through real-time prospective study designs. Further assessment of these models’ impact in diverse clinical and other population settings would be a direction that showcases the promising future of using AI and ML as a standard for remote or assisted PBM.

### Supplementary Information

**Additional file 1**. Full search strategy and study summary.

## Data Availability

All data generated or analysed during this study are included in this published article and its supplementary information file.

## Abbreviations

AI: Artificial intelligence
AIS: Abbreviated injury scale
ANN: Artificial neural network
AUROC: Area under the receiver operating curve
BBN: Bayesian belief network
BN: Bayesian network
CART: Classification and regression tree
CRI: Critical reserve index
CT: Computed tomography
DBP: Diastolic blood pressure
DNN: Deep neural network
DT: Decision Tree
ER: Evidential reasoning
FAST: Focused assessment with Sonography for Trauma
FFP: Fresh frozen plasma
FIS: Fuzzy inference system
GCS: Glasgow Coma Score
GNB: Gaussian Naïve Bayes Classifier
HR: Heart rate
ICU: Intensive Care Unit
INR: International normalized ratio
ISS: Injury severity score
KNN: k-nearest neighbor algorithm
LASSO: Least absolute shrinkage and selection operator
LDA: Linear discriminant analysis
LBNP: Low body negative pressure
LR: Logistic regression
MAP: Mean arterial pressure
MGAP: Mechanism, Glasgow Coma Score, Age and Arterial Pressure
ML: Machine learning
MLP: Multi-layer perceptron model
MT: Massive transfusion
NB: Naïve Bayes
NCCT: Non-contrast computed tomography
NLP: Natural language processing
NTDB: National trauma data bank
OCT: Optimal classification trees
PPM: Personal predictive monitoring
PR: Pulse rate
RBC: Red blood cell
RBFN: Radial-basis function network
RCT: Randomized controlled trial
RF: Random forest
RR: Respiration rate
RSNNS: Stuttgart neural network simulator
RTS: Revised trauma score
SBP: Systolic blood pressure
SCS: Simplified consciousness score
SpO_2_: Oxygen saturation
SVM: Support vector machine
TBI: Traumatic brain injury
TEG: Thromboelastography
TOP: Trauma outcome predictor
TSM: Trauma severity model
UKTRISS: United Kingdom Trauma and Injury Severity Score
