# Supplementary material for: Artificial intelligence and machine learning for hemorrhagic trauma care
Source: Mil Med Res. 2023 Feb 16;10:6. doi: 10.1186/s40779-023-00444-0 (PMC9933281; doi:10.1186/s40779-023-00444-0)
Supplement: Supplementary file 1 — Additional file 1. Full search strategy and study summary. [file 40779_2023_444_MOESM1_ESM.pdf]

Table S1 Full search strategy used for literature search

Search attempt	Combination of keywords
1	Artificial intelligence [MeSH terms] AND trauma [MeSH terms] AND bleed [MeSH terms]
2	Artificial intelligence AND trauma AND bleeding
3	Artificial intelligence AND trauma AND hemorrhage
4	Artificial intelligence AND trauma AND hemorrhaging
5	Artificial intelligence AND trauma AND haemorrhage
6	Artificial intelligence AND trauma AND haemorrhaging
7	Artificial intelligence AND trauma AND coagulopathy
8	Artificial intelligence AND trauma AND coagulopathic
9	Artificial intelligence AND trauma AND mortality
10	Artificial intelligence AND trauma AND outcome
11	Artificial intelligence AND trauma AND severe injury
12	Artificial intelligence AND trauma AND transfusion
13	Artificial intelligence AND trauma AND triage
14	Artificial intelligence AND trauma AND triaging
15	Artificial intelligence AND trauma AND soldiers
16	Artificial intelligence AND trauma AND resuscitation
17	Artificial intelligence AND trauma AND combat
18	Artificial intelligence AND trauma AND combat casualty care
19	Artificial intelligence AND trauma AND remote care
20	Machine learning AND trauma AND bleed
21	Machine learning AND trauma AND bleeding
22	Machine learning AND trauma AND hemorrhage
23	Machine learning AND trauma AND hemorrhaging
24	Machine learning AND trauma AND haemorrhage
25	Machine learning AND trauma AND haemorrhaging
26	Machine learning AND trauma AND coagulopathy
27	Machine learning AND trauma AND coagulopathic
28	Machine learning AND trauma AND mortality
29	Machine learning AND trauma AND outcome
30	Machine learning AND trauma AND severe injury
31	Machine learning AND trauma AND transfusion
32	Machine learning AND trauma AND triage
33	Machine learning AND trauma AND triaging

34	Machine learning AND trauma AND soldiers
35	Machine learning AND trauma AND resuscitation
36	Machine learning AND trauma AND combat
37	Machine learning AND trauma AND combat casualty care
38	Machine learning AND trauma AND remote care
39	AI AND trauma AND bleed
40	AI AND trauma AND bleeding
41	AI AND trauma AND hemorrhage
42	AI AND trauma AND hemorrhaging
43	AI AND trauma AND haemorrhage
44	AI AND trauma AND haemorrhaging
45	AI AND trauma AND coagulopathy
46	AI AND trauma AND coagulopathic
47	AI AND trauma AND mortality
48	AI AND trauma AND outcome
49	AI AND trauma AND severe injury
50	AI AND trauma AND transfusion
51	AI AND trauma AND triage
52	AI AND trauma AND triaging
53	AI AND trauma AND soldiers
54	AI AND trauma AND resuscitation
55	AI AND trauma AND combat
56	AI AND trauma AND combat casualty care
57	AI AND trauma AND remote care
58	ML AND trauma AND bleed
59	ML AND trauma AND bleeding
60	ML AND trauma AND hemorrhage
61	ML AND trauma AND hemorrhaging
62	ML AND trauma AND haemorrhage
63	ML AND trauma AND haemorrhaging
64	ML AND trauma AND coagulopathy
65	ML AND trauma AND coagulopathic
66	ML AND trauma AND mortality
67	ML AND trauma AND outcome
68	ML AND trauma AND severe injury
69	ML AND trauma AND transfusion

70	ML AND trauma AND triage
71	ML AND trauma AND triaging
72	ML AND trauma AND soldiers
73	ML AND trauma AND resuscitation
74	ML AND trauma AND combat
75	ML AND trauma AND combat casualty care
76	ML AND trauma AND remote care
77	Artificial intelligence AND trauma AND hemorrhage AND military
78	Artificial intelligence AND trauma AND hemorrhage AND soldiers
79	Artificial intelligence AND trauma AND hemorrhage AND transfusion
80	Artificial intelligence AND trauma AND hemorrhage AND coagulopathy
81	Artificial intelligence AND trauma AND hemorrhage AND resuscitation
82	Artificial intelligence AND trauma AND hemorrhage AND triage
77	Machine learning AND trauma AND hemorrhage AND military
78	Machine learning AND trauma AND hemorrhage AND soldiers
79	Machine learning AND trauma AND hemorrhage AND transfusion
80	Machine learning AND trauma AND hemorrhage AND coagulopathy
81	Machine learning AND trauma AND hemorrhage AND resuscitation
82	Machine learning AND trauma AND hemorrhage AND triage
83	AI AND trauma AND hemorrhage AND military
84	AI AND trauma AND hemorrhage AND soldiers
85	AI AND trauma AND hemorrhage AND transfusion
86	AI AND trauma AND hemorrhage AND coagulopathy
87	AI AND trauma AND hemorrhage AND resuscitation
88	AI AND trauma AND hemorrhage AND triage
89	ML AND trauma AND hemorrhage AND military
90	ML AND trauma AND hemorrhage AND soldiers
91	ML AND trauma AND hemorrhage AND transfusion
92	ML AND trauma AND hemorrhage AND coagulopathy
93	ML AND trauma AND hemorrhage AND resuscitation
94	ML AND trauma AND hemorrhage AND triage

Three combinations were mainly used for searches in all fields through PubMed and Google Scholar databases with the aim of finding studies using artificial intelligence (AI) and machine learning (ML) relating to some forms of traumatic hemorrhage.

Table S2 Research articles using machine learning to predict various problems in hemorrhagic trauma care

Type	Study design	AI machine learning	Prediction performance/Main findings	Reference
TO	Data from 3401 adult patients ere collected from different ICUs (surgical, trauma, coronary, cardiac) to train models for predicting mortality.<br>Data from patients younger than 18 years old was excluded.	LDA, GNB, KNN, DT (CART), and DNN model (Deep-FLAIM). Models were also compared with other mortality prediction tools.<br>Data was split into 70% training and 30% testing for CV	**AUROC** (LDA = 0.830, GNB = 0.836, KNN = 0.818, CART = 0.784, Deep-FLAIM = 0.912, ISS = 0.790, TRISS = 0.903, APACHE II = 0.770, LODS= 0.882, SOFA = 0.815, OASIS = 0.652, SAPS II = 0.786); **Accuracy** (LDA = 0.8184, GNB = 0.8007, KNN = 0.8494, CART = 0.8959, Deep-FLAIM = 0.9225); **Sensitivity** (LDA = 0.7217, GNB = 0.6783, KNN = 0.6696, CART= 0.6348, Deep-FLAIM = 0.7913, ISS = 0.639, APACHE II = 0.412, SOFA = 0.6493, OASIS = 0.6493, SAPS II = 0.627); **Specificity** (LDA = 0.8325, GNB = 0.8125, KNN = 0.8756, CART = 0.9340, Deep-FLAIM = 0.9416, ISS = 0.857, APACHE II = 0.627, SOFA = 0.785, OASIS = 0.558, SAPS II = 0.871); **PPV** (LDA = 0.396, GNB = 0.3529, KNN = 0.44, CART = 0.584, Deep-FLAIM = 0.6642); **NPV** (LDA = 0.9535, GNB = 0.9457, KNN = 0.9478, CART = 0.946, Deep-FLAIM = 0.9687);<br>Deep-FLAIM was the best model with a high accuracy on test data.<br>Features with statistical significance to the model accuracy were Sodium, Chloride, bicarbonate, creatinine, glucose, anion gap, lactate, platelets, PTT were features that showed statistical significance to trauma outcome	[1]

	Data of 129,786 patients that arrived alive to the ED was used to create models for predicting outcomes following traumatic injuries	DT, RF, ANN (Scaled conjugate gradient backpropagation, Levenberg-Marquardt backpropagation and Bayesian regularization backpropagation), SVM, LR, and Evidential Reasoning (ER) algorithms.<br>Data was split into a 10-fold CV (performed 3 times and averaged for results)	**AUROC** [DT = 0.8833, ANN (Scaled conjugate gradient backpropagation) = 0.9074, ANN (Levenberg-Marquardt backpropagation) = 0.9076, ANN (Bayesian regularization backpropagation) = 0.9068, RF = 0.9022, SVM = 0.7186, LR = 0.904, ER = 0.8797]; **Accuracy** [DT = 0.9411, ANN (Scaled conjugate gradient backpropagation) = 0.8416, ANN (Levenberg-Marquardt backpropagation) = 0.9555, ANN (Bayesian regularization backpropagation) = 0.9556, RF = 0.9428, SVM = 0.9418].<br>Levenberg-Marquardt and Bayesian Regularization backpropagated ANN algorithms provided best result	[2]
	Data of 177,014 patients that arrived alive to the ED was used to create models for predicting outcomes following traumatic injuries	Feature extractions using ER, RF, and ReliefF were done, after which SVM models were developed.<br>Data was split into a 10-fold CV (performed 3 times and averaged for results)	**AUROC** (SVM:ER = 0.895, SVM:RF = 0.885, SVM:ReliefF = 0.825).<br>Feature Selection using ER improved the AUROC of the SVM model	[3]
	Data from 1564 trauma patients were taken. Any data that did not fulfill the United Kingdom TARN (UKTARN) inclusion criteria or any missing value within the dataset was excluded	United Kingdom Trauma and Injury Severity Score (UKTRISS) was compared with an ANN model.<br>The dataset was divided into 70% training 30% testing for cross validation<br>Variables used for developing the model were AIS for each body region, RR, SBP, GCS, oxygen, saturation, and HR	**AUROC** (ANN = 0.921, UKTRISS = 0.941); Sensitivity (ANN = 0.869, UKTRISS = 0.9167); **Specificity** (ANN = 0.8571, UKTRISS = 0.9167).<br>Head injury, age and chest injury were significantly important predictors for provided a better model fit	[4]

	Data from 2,007,485 trauma patients with complete ED vitals and a valid outcome was used.	Different XGBoost models were created with different age demographics (children, adults, all ages).<br>Variables employed to develop the model were from the following categories: ED vitals, injury type, injury mechanism, comorbidities, arrival mode, arrival status and outcome.<br>The dataset was divided into 70% training 30% testing for cross validation	**AUROC** [child model = 0.91, adult model = 0.89, all ages model = 0.90]; **Prevision = PPV** [child model = 0.09, adult model = 0.12, all ages model = 0.10]; **NPV** [child model = 0.998, adult model = 0.993, all ages model = 0.995]; **Sensitivity** [child model = 0.80, adult model = 0.79, all ages model = 0.84]; **Specificity** [child model = 0.92, adult model = 0.84, all ages model = 0.80].<br>XGBoost as a model showed a high AUROC for all age groups. It was noted that excluding fall injuries lowered the AUROC for the children model, but increased the AUROC for the adult model. The model involving all age groups was unchanged	[5]
	Data from 1658 trauma Audit patients treated at the North Staffordshire Hospital were taken. Predicting whether a trauma patient will die given the various factors such as type of trauma incident, length of stay (LOS) etc.	Artificial NN (ANN) performed with 5-fold cross validation. Logistic regression modelling was performed using the dataset.	**Accuracy** (ANN = 0.8, LR = 0.77); **Precision = PPV** (ANN = 0.55, LR = 0.49); **NPV** (ANN = 0.89, LR = 0.92).<br>While the ANN overall predictive accuracy is better than that of the logistic regression model, the logistic regression model is better at predicting death than all 4 ANN models	[6]
	Cohort data from 1494 severely injured trauma patients from emergency department admissions was used. Patients that met criteria for the highest triage activation level were included, whereas patients less than 15 years, pregnancy, incarceration, transfer from outside hospital were excluded.<br>Data with missing outcomes was excluded.	Ensemble Machine learning Algorithm (SuperLearner) trained with data collected at admission, 2, 3, 4, 6, 12, 24, 48, 72, 96, 120 h after injury. Performance was measured for different variables to assess variable importance.<br>5-fold CV was employed for cross validation	**AUROC** (death = 0.94 − 0.97, multi-organ failure = 0.84 − 0.90, transfusion = 0.87 − 0.90, acute respiratory distress syndrome = 0.84 − 0.89, venous thromboembolism = 0.73 − 0.83, coagulopathic trajectory = 0.48 − 0.88);<br>SuperLearner fit showed an excellent performance using the variables death, multi-organ failure, and transfusion	[7]
	Dataset from 64 patients requiring damage control surgery.<br>Any data with missing feature values was removed	Decision tree (using recursive partitioning algorithm), Naive Bayes Classifier.<br>Features that were used to train the models were patient Characteristics, features of prehospital care, physical and lab findings in ER, operating room and ICU.<br>10-fold CV was conducted	**AUROC** (DT = 0.849, NB = 0.882); **Accuracy** (DT = 0.824, NB = 0.794); **Sensitivity** (DT = 0.822, NB = 0.8); **Specificity** (DT = 0.696, NB = 0.826).<br>NB classification model performed better based on a higher	[8]

	Cohort data of 260,505 trauma patients aged 65 years and older were included in validating a Trauma outcome predictor (TOP) by Maurer et al. for geriatric cases	TOP model is a developed using an optimal classification tree algorithm.<br>Relevant features that were used from the data included patient demographics, comorbidities, type and mechanism of injury, Injury Severity Score (ISS), AIS, pre-hospital and emergency department (ED), vital signs, diagnoses, complications, and mortality	Results for specific outcomes using the TOP algorithm **AUROC** (Acute respiratory distress syndrome = 0.75, cardiac arrest = 0.75, deep surgical site infection= 0.95, organ space surgical site infection = 0.84).<br>Results for these specific outcomes using TOP highlight high predictive performance	[9]
	Retrospective data from prehospital, emergency of 10,609 trauma patients from January 1993 to December 1996 was used	Feed-forward back-propagation NN (ANN) was developed. The model's performance was compared with Trauma and Injury Severity Score (TRISS)-MTOS derived coefficient, and Injury Severity Score (ISS).<br>Input Variables used: GCS, SBPHR, RR, temperature, hematocrit, age, sex, intubation status, ICD-9-CM Injury E-code, and ISS.<br>Data was divided throughout the years. The models and the scoring system were trained using the 1993-1994/1995 datasets and tested using the 1995/1996	**AUROC** (ANN = 0.912, TRISS = 0.895, ISS = 0.766). ANN showed good data clustering, and good separation between survivors and non-survivors. The ANN model surpassed TRISS in non-survivor predictability.<br>Implementation of ISS and GCS as features increased the AUROC of the ANN model (0.891 without GCS and 0.894 without ISS)	[10]
	Two Dataset were used to train the model - the National Trauma Data Bank (NTDB) and the Nationwide Readmission Database (NRD). Risk prediction was identified as a binary outcome (alive or dead before discharge).<br>Patients were included on the following criteria: (1) patient had at least one ICD-9 code between 800-959.9 recorded during hospital visit, (2) Patient admission must be an emergency admission with valid E-code, (3) patient discharge status was not due to transfer to a different ED	Machine learning model - Trauma Severity Model (TSM), compared with other risk prediction model: Bayesian Logistic Injury Severity Score (BLISS), Harborview Assessment for Risk of Mortality (HARM), and the Trauma Mortality Prediction Model (TMPM). TSM is a stacked generalization algorithm (ensemble algorithm using NN, RF, GBM methods).<br>Two sets of models were created - one using the IC-9 codes as input variables, the other using IC-9, patient demographics, general trauma assessments as input variables (augmented models).<br>Cross validation was done using the out-of-sample validation method	Models developed using ICD-9 codes: **AUROC** (TSM = 0.912, BLISS = 0.900, HARM = 0.866, TMPM = 0.898); **Accuracy** (TSM = 0.968, BLISS = 0.967, HARM = 0.965, TMPM = 0.966); **F-measure** (TSM = 0.404, BLISS = 0.369, HARM = 0.299, TMPM = 0.336).<br>Augmented models: **AUROC** (TSM = 0.965, BLISS = 0.957, HARM = 0.955, TMPM = 0.958); **Accuracy** (TSM = 0.976, BLISS = 0.975, HARM = 0.973, TMPM = 0.973); **F-measure** (TSM = 0.621, BLISS = 0.601, HARM = 0.564, TMPM = 0.573).<br>TSM performed very well considering everything.<br>Statistically Significant variables: ICD-9 models - intracranial hemorrhage, subdural hemorrhage and concussion Augmented - GCS eye score of 1, GCS verbal score, patient’s age.<br>Variables pertaining to the head trauma was an important information in predicting patient outcomes	[11]

	A database containing records of 15,055 trauma patients was used to improve the TRISS method to assess the probability of survival of patients admitted to trauma units.<br>If the outcome variable was missing then data was discarded	The Trauma Injury Severity Score (TRISS), a model that re-optimized TRISS coefficients (re-optimized TRISS), LR, and Multilayer Perceptron NN models were implemented.<br>Two LR model were developed: (1) using age, revised trauma score (RTS), ISS and injury type and (2) using age, revised trauma score (RTS), ISS.<br>A 50:50 training test data split was implemented for cross validation	**AUROC** [TRISS = 0.9411, Re-optimized TRISS = 0.9426, LR (1) = 0.9534, LR (2) = 0.9521, NN = 0.9548]	[12]
	Data of 628 patients from a trauma patient dataset were randomly selected for a model	A feed-forward, back propagation NN model was developed with a total of 186 input layers.<br>Variables from the following categories were utilized in training the model: location of Trauma, Injury etiology, respirator/ventilator assistance	**Accuracy** = 0.91; **Sensitivity** = 0.78; **Specificity** = 0.94; **PPV** = 0.73; **NPV** = 0.95	[13]

	Data pertaining to the last 150 trauma patients at a surgical ICU is used for model training and evaluation. The aim of the study was to evaluate the importance of resuscitation and its impact on mortality prediction.	A fuzzy logic inference system is developed, which includes SBP, GCS, and changes after 1hr of resuscitation. This system is compared with common scoring methods such as ISS, RTS, ASCOT and TRISS [(1) using coefficients from Major Trauma Outcome Study (MTOS) and (2) using coefficients from the National Trauma Data Bank (NTDB)]	At ER arrival: **AUROC** [RTS = 0.811, TRISS (1) = 0.899, TRISS (2) = 0.886, ASCOT = 0.886]; Accuracy [RTS = 0.787, TRISS (1) = 0.86, TRISS (2) = 0.873, ASCOT = 0.82]; **Precision = PPV** [RTS = 0.706, TRISS (1) = 0.821, TRISS (2) = 0.885, ASCOT = 0.75]; **NPV** [RTS = 0.797, TRISS (1) = 0.869, TRISS (2) = 0.871, ASCOT = 0.833].<br>At 1 h: **AUROC** [RTS = 0.916, TRISS (1) = 0.978, TRISS (2) = 0.967, ASCOT = 0.961]; **Accuracy** [RTS = 0.907, TRISS (1) = 0.913, TRISS (2) = 0.92, ASCOT = 0.887]; **Precision = PPV** [RTS = 0.879, TRISS (1) = 0.906, TRISS (2) = 0.886, ASCOT = 0.923]; **NPV** [RTS = 0.915, TRISS (1) = 0.915, TRISS (2) = 0.93, ASCOT = 0.879].<br>At ICU: **AUROC** [RTS = 0.903, TRISS (1) = 0.968, TRISS (2) = 0.966, ASCOT = 0.96]; **Accuracy** [RTS = 0.92, TRISS (1) = 0.92, TRISS (2) = 0.927, ASCOT = 0.913]; **Precision = PPV** [RTS = 0.88, TRISS (1) = 0.886, TRISS (2) = 0.912, ASCOT = 0.93.3]; **NPV** [RTS = 0.93, TRISS (1) = 0.93, TRISS (2) = 0.931, ASCOT = 0.908]; **ISS** (AUROC = 0.903, Accuracy = 0.88, PPV = 0.818, NPV = 0.897); **Fuzzy Logic** (AUROC = 0.925, Accuracy = 0.86, PPV = 0.781, NPV = 0.881).<br>The response to resuscitation has a significant impact on the mortality prediction.	[14]
	Trauma patient data from the NTDB was extracted to develop an automated decision-making algorithm for remote triaging. Patients who suffered blunt or penetrating trauma injuries were included. Missing value data was excluded	LR, RF, DNN models were developed, using HR, SBP, RR, age, simplified consciousness score (SCS) and/or GCS as the input variables. The models were also compared with other trauma scoring methods like RTS and TRISS.<br>10-fold validation was implemented as a cross-validation method	**AUROC** (RTS = 0.78, LR = 0.88, RF = 0.87, DNN = 0.89, TRISS = 0.90).<br>SCS was the most important feature observed for survival prediction, and SBP was the second most important.<br>The DNN model (with SCS as the input variable) performed best out of all three models	[15]

	Data from 1190 trauma patients who were directly sent to the ED after an accident was used. Data was included based on the following criteria: (1) the five vital signs were recorded upon ED arrival (2) corresponding in-hospital data was retrievable. Missing data values were excluded	Rule-based inference methodology using the evidential reasoning approach (RIMER) was used to develop a decision model to predict probability of in-hospital death. RIMER was compared with other commonly used methods, such as SVM, ANN, and LR. Relevant variables for model development were pulse rate (PR), SBP, RR, Temperature, Consciousness.<br>5-fold validation was implemented as a cross-validation method	**AUROC** (RIMER = 0.952, LR = 0.885, SVM = 0.821, ANN= 0.79);<br>Out of the three, LR shows a statistically significant correlation for an outcome. However, the RIMER model outperforms the three models	[16]
	Trauma Data collected from the Hellenic Trauma and Emergency Surgery Society (HTESS) to develop models for trauma outcome prediction. Patients who arrived dead or died at the ER were excluded	Multi-layer Perceptron (MLP), Radial Basis Function NNs (RBFN), Classification and Regression Trees (CART), and compared with LR. The metrics of the two NNs (MLP and RBFN) are mentioned together.<br>Variables used to train were: weight, age, GCS, PR, SBP, DBP, Hematocrit, haemoglobin, White cell count, glucose, creatinine, amylase, ISS, RTS.<br>The data was split 50% for training, 25% for testing and 25% for cross validation	**AUROC** (NN = 0.887, LR = 0.913, TRISS = 0.682, RTS = 0.641); **Accuracy** (CART = 0.9897, NN = 0.992, LR = 0.989, TRISS = 0.98864, RTS = 0.98641); **Sensitivity** (CART = 0.1, NN = 0.83, LR = 0.648); **Specificity** (CART = 0.9892, NN = 0.993, LR = 0.995); **PPV** (CART = 0.7889, NN = 0.43, LR = 0.685); **NPV** (CART = 0.1, NPV = 0.999, LR = 0.994).The NN models showed a high accuracy and AUROC, and outperformed the other models	[17]
	Data from motorcycle riders who were hospitalized was collected to develop ML models for mortality prediction of motorcycle riders. Only patients who sustained injuries from motorcycle accidents were included	SVM, DT (CART) models were developed to predict mortality outcome of these subjects. Relevant features included sex, whether a helmet was worn, AIS, GCS, ISS.<br>10-fold validation was implemented as a cross-validation method	**AUROC** (SVM = 0.9534, LR = 0.9528, DT = 0.8872); **Accuracy**(SVM = 0.9862, LR = 0.9864, 0.9892); **Sensitivity** (SVM = 0.6207, LR = 0.5931, DT = 0.6276); **Specificity** (SVM = 0.9948, LR = 0.9956, DT = 0.9977).<br>All models had a high accuracy, although SVM had the higher sensitivity even when sample set was reduced. Both LR and SVM had a high AUC, in mortality prediction	[18]
	Data from 2232 patients with severe trauma injuries was used in building an automated decision-based early prognostic model based on admission. Patients > 16 years old were included into the cohort	An XGBoost algorithm was used to build the model as it was seen to have the highest accuracy.<br>GCS, HR, RR, MAP prehospital cardiac arrest, AIS of head and neck, thorax, and abdomen, and ED interventions.<br>The data was split 80:20 Training: Test/validation	**Accuracy** = 0.94; **Sensitivity** = 0.98; **Specificity** = 0.548; **PPV** = 0.954; **NPV** = 0.742.<br>The XGBoost model had a high accuracy, sensitivity and PPV at predicting mortality. Only features that could be determined within the first 2 h of admission were used	[19]

	All patients in the ACS-TQIP database were used. Patients who died in the emergency department were excluded.<br>Missing data was imputed using the ML method Optimal Impute	Optimal Classification Trees (TOP) was developed and validated.<br>An 80:20 training: validation split was done to the dataset. Models for blunt and penetrating trauma were developed and compared.<br>Variables used for model development were demographics, vital signs (SBP, HR, RR, SpO_2, GCS, temperature), comorbidities, and injury characteristics	**AUROC** [TOP (B) = 0.88, TRISS(B) = 0.866, TOP(P) = 0.941, TRISS(B) = 0.935].<br>The TOP algorithm was accurate at predicting mortality in penetrating and blunt injuries and has a higher performance than TRISS models	[20]
	Retrospective study that used the data of 7688 trauma patients admitted to the Swedish Medical Center	NN model designed using five input vars and one output. RTS and RPS scores were used for comparison.<br>Significant variables were RTS, GCS, SBP, RR, PR	**Accuracy** = 0.91.<br>Performance was too sensitive to require refinements. RTS and RPS was not effective as they had low sensitivity and low specificity	[21]
	Data of 116,000 trauma patients (patients with an ICD-9-CM score between 800 and 959.9) were obtained from the North Carolina Medical Database (NCMD).<br>The inclusion criteria involved patients admitted to the hospitals in North Carolina for at least 1 d	A Predictive Hierarchical Network Model was developed. ISS derived using the technique described by MacKenzie Abbreviated Injury Score (AIS), was used to compare the network.<br>The database of 116,000 patients was randomly split into training and evaluation subsets.<br>Relevant features used: AIS and body system maximum AIS scores, mortality risk ratios derived from the ICD-9-CM primary, secondary, and tertiary diagnoses, primary and secondary procedures as described in previous work, age and gender	**Accuracy** (Network = 0.983, ISS = 0.90); **Sensitivity** (Network = 0.994, ISS = 0.919, TRISS = 0.992, 0.993);<br>**Specificity** (ISS = 0.455, Network = 0.502, TRISS = 0.588, ASCOT = 0.633).<br>The network was the better predictor of outcome than the ISS, and was almost as accurate, sensitive, and specific as reported values for TRISS and A Severity Characterization of Trauma (ASCOT) without access to physiologic information	[22]

	Patient data from the North Carolina Trauma Registry (NCTR) with an International Classification of Diseases Supplementary Classification of Diagnosis score (ICISS) between 800 and 959.9 were used.<br>All patients with missing values or incomplete results were excluded	A special type of NN, known as polynomial neural nets, was used, and was compared with other trauma ranking scores like ISS, RTS, TRISS, and ICSS.<br>The data was divided into training and testing dataset	**AUROC** (ISS =0.667, TRISS = 0.877, ICSS = 0.916, RTS = 0.95, NN = 0.98); **R-squared** (ISS = 0.0849 for hospital charges/0.052 for hospital length of stay, TRISS = 0.1449 for hospital charges/0.0559 for hospital length of stay, ICSS = 0.5116 for hospital charges/0.2499 for hospital length of stay, RTS = 0.5353 for hospital charges/0.2974 for hospital length of stay, NN = 0.7521 for hospital charges/0.5345 for hospital length of stay).<br>ICISS-derived predictions of survival, hospital charges, and hospital length of stay consistently outperformed those of ISS and TRISS. The neural network- augmented ICISS was even better	[23]
	Patient data from the Royal Melbourne Hospital (RMH). Blunt trauma cases from the Victorian State Trauma Registry (VSTR) were used for validation.<br>Patients with complete data are included. Penetrating trauma cases were excluded	LR, DT using recursive Partitioning, ANN were developed by different authors. All authors were blinded to the validation dataset when developing models.<br>Age, SBP, RR and pulse rate, Injury severity score, GCS were relevant features for model development	Performance for ICU stay/survival: **AUROC** (LR = 0.79/0.91, ANN = 0.78/0.83); **Precision = PPV** (0.37/0.18, DT = 0.44/0.15, ANN = 0.39/0.14); **NPV** = 0.92/0.99, DT = 0.86/0.99, ANN = 0.90/0.98); **Sensitivity** (LR = 0.90/0.77, DT = 0.70/0.61, ANN = 0.84/0.70); **Specificity** (LR = 0.46/0.83, DT = 0.68/0.85, ANN = 0.52/0.80).<br>Performance of the three models for ICU stay was similar; Logistic regression performed slightly better than the other two methods for survival prediction	[24]
	Study data was taken from the NTDB.<br>Missing values were excluded	Multilayer perceptron (MLP) methodology was used to create ANN model algorithms.<br>85% of the data (1,217,125) were randomly selected as a training set, and 15% (215,899) were used as the test set.<br>SBPRR, AIS Ability to obey simple commands and age were features used for model development	**RMSE** (ANN = 0.1999)<br>Models performed well in predicting mortality compared to standard outcome predictors (Revised Trauma Score and the TRISS Probability of Survival)	[25]

	Study data was taken from the NTDB dataset to develop models to predict complications during patient hospitalization	Tiberius Software created the ANN model.<br>No ventilation/intubation, in ICU ward for 2+ d, SBPRR, Age, Sex with an ICU bed for more than 2 d were most important features for model development	**RMSE** (ANN for ARDS = 13.84, ANN for VAP = 14.42, ANN for UTI = 26.39);<br>The basic ANN is accurate for those likely to contract ARDS though with a high rate of false positives. The ANN ability to predict VAP is less effective, though better at producing fewer false positives. Predicting UTI cases is not good	[26]
	Study participants were recruited from wounded US service members evacuated from Iraq and Afghanistan.<br>Adult, active duty service members who sustained penetrating extremity injuries during combat operations abroad were included.<br>Participants with pre-morbid confounding inflammatory conditions, including immune deficiency and connective tissue disorders, or any medical illness requiring immunosuppressive therapy were excluded	These prospective studies analyzed various combat casualties using stepwise machine-learned BBN and step-wise LR.<br>Models were evaluated using 10-fold cross-validation.<br>Likelihood of nosocomial infection can be estimated a priori using serum albumin, injury severity score (ISS), and initial transfusion requirement; impaired wound; Healing can be estimated a priori using ICU admission; and likelihood of ICU admission can be estimated using initial transfusion requirement and Acute Physiology and Chronic Health Evaluation II (APACHE II) score. In the second step of each equation, four biomarkers were entered: (Interleukin) IL-6, IL-8, IL-12 p40, and MCP1	Performance for models with biomarkers for impaired wound healing/ICU admission/nosocomial infection: **AUROC** (LR = 0.91/0.97/0.81, BBN = 0.71/0.81/0.79).<br>Performance for models without biomarkers for impaired wound healing/ICU admission/nosocomial infection: **AUROC** (LR = 0.91/0.97/0.81, BBN = 0.79/0.76/0.76); **Accuracy** (LR = 0.857/0.93/0.73, BBN = 0.767/0.80/0.76); **Precision = PPV** (LR = 0.10/0.87/0.55, BBN = 0.40/0.72.7/0.75); **NPV** (LR = 0.81/0.99/0.84, BBN = 0.95/0.842/0.682); **Sensitivity**(LR = 0.61/0.98/0.67, BBN = 0.80/0.727/0.538); **Specificity** (LR = 0.10/0.93/0.75, BBN = 0.76/0.842/0/882).<br>BBN model is indeed performed as robust and these results were re-affirmed by stepwise LR modeling using features identified by the BBN	[27]
	Patients recorded in the NTDB with one or multiple injuries with filled screening tests were used.<br>Missing or out-of-range values were excluded	BDT models for 1-20 injuries were developed and compared with TRISS.<br>The most important contribution is made by age, thorax severity, and BP. By contrast, gender, Glasgow Eye Coma Score, and neck severity are least important or redundant	**AUROC** (BDT = 0.954, TRISS = 0.948); **Accuracy** (BDT = 0.971, TRISS= 0.968); **Sensitivity** = (BDT = 0.474, TRISS = 0.528); **Specificity** (BDT = 0.994, TRISS = 0.988).<br>The Bayesian Decision Tree model outperforms the TRISS model in terms of goodness-of-fit and classification accuracy	[28]

	Consecutive patients admitted to an urban Level I Trauma Center were used to develop models for mortality prediction of trauma patients.<br>Patients were excluded if they have only one time point with measurements	LR with elastic net regularization was used to accommodate large numbers of missing values as well as highly correlated time-course data.<br>Data was randomly split into 80% training data and 20% testing data for evaluation.<br>Features were excluded if they have ≥30% missing values across all included measurements	**AUROC** = 0.67; **TPR** = 0.66; **TNR** = 0.89.<br>Temporal importance-adjustment model for predicting mortality, which incorporates both time-dependent and missing value indicator functions in a logistic regression model with elastic net regularization	[29]
	Study data was taken from a trauma management report with admission dates 2005 through 2006.<br>Patients who arrived dead or died at the ER of each hospital were excluded from the analysis.<br>Missing data values was handled using Multiple imputation (MI)	Two NN algorithms were tested: MLP and RBFN. These two models were compared with LR, TRISS and RTS.<br>Dataset was split 50% training: 25% testing: 25% validation.<br>Pearson's chi-square statistical test was used for feature selection	**AUROC** (NN = 0.869, LR = 0.922, RTS = 0.634, TRISS = 0.671); **Accuracy** (NN = 0.9902, LR = 0.986); Precision (NN = 0.40, LR = 0.333); **NPV** (NN = 0.998, LR = 0.996); **Sensitivity** (NN = 0.80, LR = 0.555); **Specificity** (NN = 0.991, LR = 0.99).<br>A comparison was also made between the abilities of TRISS and RTS to predict fatalities showing that the ANN (MLP) outperformed the logistic regression at correctly classifying cases in a training, testing and validity test	[30]
	The records of almost 2 million patients hospitalized with traumatic injuries from the US National Trauma Data Bank (NTDB) was used.<br>Records having missing or out-of-range values, or patients having no ISS were excluded	Various models are developed for predicting outcome: KNN, unpruned C4.5 DT, multinomial LR model with ridge estimator, NB classifier, ANN, PART decision list, RF, TRISS, SVM with SMO.<br>Seventeen different features were used- 15 numerical and 2 nominals (gender and injury type)	**AUROC** (KNN = 0.8103, DT = 0.90173, LR = 0.94638, NB = 0.93008, ANN = 0.95122, PART = 0.92654, RF = 0.95352, TRISS = 0.94907, SVM = 0.93061); **Accuracy** (KNN = 0.968, DT = 0.9726, LR = 0.9693, NB = 0.9397, ANN = 0.9718, PART = 0.971, RF = 0.9774, TRISS = 0.9647, SVM = 0.9693); **Sensitivity** (KNN = 0.9848, DT = 0.9922, LR = 0.9925, NB = 0.9503, ANN = 0.9936, PART = 0.9919, RF = 0.9944, TRISS = 0.9812, SVM = 0.9968); **Specificity** (KNN = 0.6042, DT = 0.5479, LR = 0.467, NB = 0.7101, ANN = 0.4992, PART = 0.5189, RF = 0.6101, TRISS = 0.6072, SVM = 0.3758); RF can outperform the TRISS methodology	[31]
	Blunt and penetrating trauma injuries cases (n = 47,702) from TARN were used to develop the FIS model	The FIS analysis examined the manner single and multiple trauma injury factors that influenced the probability of survival.<br>Age and sex, AIS and GCS values, BP, HR, RR	The fuzzy logic compares injury information about a case with those in the database to determine the likelihood of the survival.<br>Work is currently ongoing to complete the FIS knowledge base	[32]

	The participants are 20,207 adult trauma patients with, or at risk of significant bleeding who were generally within 8 h of injury.<br>Missing values or constant values were replaced with *null*	LR and NB models are developed and compared for mortality/ICU admission prediction.<br>10-fold cross-validation was used. The data is split into 90:10 training and testing sets and repeated for each of the 10-folds.<br>age, GCS, SBP, RR, capillary refill time, HR were used for model development	Model performance for mortality/ICU admission: **AUROC** (LR = 0.8316/0.7429, NB = 0.7524/0.7275, NN = 0.8176/0.7558, RF = 0.8254/0.7818).<br>There is no clear winner between the presented prediction models	[33]
	Dataset of 18,811 trauma patients was used in the retrospective study.	The data is split into 70:30 training and testing sets.<br>Thirty-one variables were used for imputation in the ML classifiers	**AUROC** (LR = 0.958, SVM = 0.964, NN = 0.944, TRISS = 0.93); **Accuracy** (LR = 0.978, SVM = 0.978, NN = 0.975, TRISS = 0.976); **Sensitivity** (LR = 0.993, SVM = 0.992, NN = 0.986, TRISS = 0.989); **Specificity** (LR = 0.385, SVM = 0.408, NN = 0.515, TRISS = 0.415).<br>TRISS exhibited significantly worse performance in predicting survival than the three remaining models	[34]
	Trauma data of 316 patients were collected from the Royal London Hospital	BA was used as a principled approach to optimal classifier systems using Decision Tree technology in which a confidence rating can be associated with every predicted result.<br>The data is split into 65:35 training and testing sets.<br>Sixteen features: age, gender, Injury, Head injury, Facial injury, Chest injury, Abdominal injury, Limbs, External injury, RR, SBP, GCS (eye, motor, verbal), Oximetry, HR	all the included techniques have nearly the same misclassification rates within 5-fold cross-validation when comparing archetypal DTs (ADT) with the Bayesian averaging (BA) and the maximum a posteriori (MAP) techniques	[35]
	Prospective data collection, level I trauma centre were included.<br>Patients admitted with complete data were included.<br>Patients with missing prediction data for TRISS calculation or WATSON criteria, as well as polytrauma patients without temperature taken before the shock room were excluded	the WATSON-based visual analytics tool for polytrauma patients is based on a local trauma data bank.<br>Age, temperature, ISS, and presence of head injury by the GCS.<br>The WATSON Trauma Pathway Explorer could act as a supporting tool in clinical decision-making. However, the visual analytics tool is not meant to be a piece of advice, and no clinical recommendation about the current patient is made	**AUROC** (WATSON for SIRS/Sepsis/Early death = 0.77/0.71/0.90).<br>The goodness of fit of WATSON was superior to that of TRISS based on Brier score (0.06 vs. 0.11 points)	[36]

	Intracranial hemorrhage cases of 248,536 patients in the National Trauma Data Bank (NTDB) from 2012 to 2016 were collected<br>Patients with a diagnosis code associated with Traumatic intracranial hemorrhage (tICH) are included.<br>Records with missing data were excluded	Linear SVM with recursive feature elimination (RFE), LR, DTC, KNN, NB, LDA were developed and compared.<br>The data split into 80:20 training testing sets. 10-fold cross validation implemented.<br>demographic information, SBP blood alcohol level (BAL), GCS, ISS, presence of epidural/subdural/subarachnoid/ intraparenchymal hemorrhage, comorbidities, complications, trauma center level, and trauma center region	**Accuracy** (linear SVM with RFE = 0.827, LR = 0.801, DTC = 0.792, KNN = 0.81, NB = 0.744, LDA = 0.812); **AUROC** (Linear SVM = 0.831); Precision (Linear SVM = 0.309); **Sensitivity** (Linear SVM = 0.75); **Specificity** (Linear SVM = 0.831).<br>In comparing the performance of a logistic regression, linear SVM, performance was highest with the linear SVM model	[37]
	968,665 unique patient data were collected from the NTDB.<br>Patients with more than 2 feature sets missing were excluded.<br>An iterative imputation method was used to impute the missing values	The data was split into 85:15 sets.<br>Twenty-two features, a number further reduced to only 8 features via the permutation importance method. Importantly, the 8 features can all be readily determined at admission: SBPHR, RR, temperature, oxygen saturation, gender, age and GCS	Gradient Boosting model: **AUROC** (comorbidities/no comorbidities = 0.931/0.924); **Accuracy** = 0.924.<br>The gradient boosting method exhibited a ROC-AUC that exceeded that of various NNs and other machine learning models	[38]
	1,611,063 adult patients from NTDB were used for model development.<br>Patients with codes for burns, drowning/submersion, environmental or exertional injuries were excluded to maintain homogeneity. Patients transferred to another hospital or missing survival information, were excluded	XGBoost, LR models were developed and compared with ISS, TMPM, ICD10 scores.<br>The data was split into 50:50 sets, 10-fold cross validation was implemented.<br>Major laceration of heart with hemopericardium and Major laceration of abdominal aorta were important features	**AUROC** (XGBoost = 0.863, LR = 0.845, ISS = 0.828, TMPM-ICD10 = 0.861); **Precision** (XGBoost = 0.895, LR = 0.819, ISS = 0.509, TMPM-ICD10 = 0.852); **Sensitivity** (XGBoost = 0.308, LR = 0.295, ISS = 0.101, TMPM-ICD10 = 0.300);<br>XGBoost demonstrated superior performance and calibration compared to logistic regression, ISS and TMPM-ICD10	[39]
	Trauma patient data from the NTDB were used to develop and compare ML model.<br>Patients with one or more injuries were included.<br>Patients with missing data were excluded	BDT models versus TRS. BDT implemented with Markov chain Monte Carlo.<br>Age, Gender, Injury type, BPRR, GCS Eye, GCS erbal, GCS Motor, Head severity, Face severity, Neck severity, Thorax severity, Abdomen severity, Spine severity, Upper extremity severity, Lower extremity severity, External severity were important features	**AUROC** (BDT = 0.954, TRS = 0.948); **Accuracy** (BDT = 0.971, TRS = 0.68); **Sensitivity** (BDT = 0.474, TRS = 0.528); **Specificity** (BDT = 0.994, TRS = 0.988);<br>BDT has outperformed TRS in terms of prediction accuracy	[40]

	Rates of amputation performed because of nonviable limb tissue was investigated using trauma patient data from the US joint trauma and UK-JTTR	A 10-predictor BN prognostic model was developed using data from the US joint trauma and was compared to the mangled extremity severity score (MESS).<br>Prognostic performance was estimated by 10-fold cross-validation in the internal validation dataset. Performance in new participants was externally validated using data from the UK-JTTR	**AUROC** (BN = 0.97, MESS = 0.70); **Sensitivity** (MESS = 0.539); **Specificity** (MESS = 0.774);<br>The BN had significantly better performance than MESS at predicting the outcome of limb revascularization	[41]
	Trauma data from the Mayo Clinic Intensive Care Unit (ICU) consisting of 23,744 ICU admissions and 18,349 unique patients were used	Four ML algorithms were considered for the development of the Trauma Triage Treatment and Training Decision Support (4TDS): random forests, logistic regression, support vector machines, and gradient boosting.<br>HR, RR, SpO_2, Non-invasive BP, Arterial BP, temperature were important features used	4TDS: **PPV** = 0.25; **NPV** = 0.97; **Sensitivity** = 0.73; **Specificity** = 0.80<br>The machine learning algorithm based on LR performed best among other algorithms we tested and was able to predict shock onset 90 minutes before it occurred with better than 75% accuracy in the test dataset	[42]
	A retrospective analysis of a trauma registry was used to identify patients admitted to a level 1 trauma center for >24.<br>Patients with no EDI values before discharge were excluded	EDI was constructed from 125 objective patient measures within the electronic health record.<br>External validation using EHR from a trauma registry OF 1,325 patients admitted to a level 1 trauma center.<br>EDI include but are not limited to age, SBPHR, RR, oxygen saturations, oxygen requirement, cardiac rhythm, blood pH, sodium, potassium, blood urea nitrogen, white blood cell count, hematocrit, platelet count, and neurologic assessments including GCS	Performance after 24 h for mortality/unplanned ICU admission: **AUROC** (Max EDI = 0.98/0.52, EDI slope = 0.85/0.66, ISS = 0.89, NISS = 0.91); Precision (Max EDI = 0.23, EDI slope= /0.86); **NPV** (Max EDI = 0.99, EDI slope = 0.02); **Sensitivity** (Max EDI = 0.93, EDI slope = 0.06); **Specificity** (Max EDI = 0.94, EDI slope = 0.60);<br>EDI Index appears to perform strongly in predicting in-patient mortality similarly to ISS and New ISS (NISS). In addition, it can be used to predict unplanned ICU admissions	[43]

	U.K. military service personnel who had sustained a perineal injury over an 8-year period were used in the development of the classifier model	A NB classifier model was built using optimal anatomical and physiological parameters. The classifier was compared against the performance of ISS, NISS, TRISS, and RTS.<br>10-fold cross validation was implemented<br>The physiological variables included the following: SBP and DBP, HR, RR, oxygen saturations, temperature, and white cell count. The severity of anatomical injuries in the pelvis, penis, testes, scrotum, and anorectum	**AUROC** (NB = 0.906, ISS = 0.844, NISS = 0.88, TRISS= 0.859, RTS = 0.851); **Accuracy** (NB = 0.906, ISS = 0.842, NISS = 0.882, TRISS = 0.859, RTS = 0.848); **Precision = PPV** (NB = 0.894, ISS = 0.858, NISS = 0.891, TRISS = 0.784, RTS = 0.788); **Sensitivity** (NB = 0.909, ISS = 0.80, NISS = 0.857, TRISS = 0.954, RTS = 0.929); **Specificity** (NB = 0.903, ISS = 0.879, NISS = 0.905, TRISS = 0.761, RTS = 0.774).<br>The NB model significantly out-performed Injury Severity Score (ISS), Trauma ISS, New ISS, and the Revised Trauma Score in virtually all areas	[44]
RA/IS	Data from 262 patients from a trauma vitals database was taken.<br>Data was included if patient suffered both blunt and penetrating injuries	Commercially available Feed-forward back propagation ANN model.<br>10-fold cross-validation was implemented	**AUROC** (ANN = 0.868)	[45]
	Patient data from 73 soldiers with extremity wounds for cases of nosocomial pneumonia	RF using BE and Logistic Regression (LR) models were developed. 2 sets of variables were chosen using Backward elimination (BE): (1) Injury Severity Score (ISS), AIS chest, and cryoprecipitate given within the first 24 h; (2) FGF-basic, IL-2R, and IL-6.<br>Leave-one-out CV was employed to test the accuracy of the model.<br>Forty-four variables were collected to train the dataset: AIS for different body regions, ISS, Transfusion of blood variables (RBC, WBC, platelets, fresh frozen plasma, cryoprecipitate)	**AUROC** (RF with variable set 1 = 0.97, LR with variable set 1 = 0.86, RF with variable set 2 = 0.87, LR with variable set 2 = 0.75); **NPV** (RF with variable set 1 = 1.0, LR with variable set 1 = 0.89, RF with variable set 2 = 0.78, LR with variable set 2 = 0.73).<br>**Sensitivity** (RF with variable set 1 = 0.89, LR with variable set 1 = 0.87, RF with variable set 2 = 0.97, LR with variable set 2 = 0.76).<br>RF algorithm using the variable set 1 (ISS, AIS chest, and cryoprecipitate given within the first 24 hours) presented an AUROC higher than both LR cases	[46]
	A cohort of 104 patients that were transferred to a level 1 trauma center in Houston Texas.<br>Vitals, GCS, and HRC of the patients were taken for further analysis and model training for prediction of LSI	Multivariate Logistic Regression (including GCS and excluding), perceptron model (using mean HR, GCS score, HRC as inputs) models were developed	**AUROC** (LR including GCS = 0.94, LR excluding GCS = 0.81, Perceptron model = 0.99).<br>Perceptron model yielded the best results and had improved performance than the multivariate LR models	[47]

	A cohort of 79 prehospital patient records were taken from the TV database.<br>The data was included based on three criteria's: (1) Availability of vital signs and patient status summary scores, (2) BP measurements over a minimum of 15 min, (3) HR measurements uncorrupted by electromechanical noise	Multilayer perceptron (activation function= sigmoid) (MLP), logistic regression, ANN, DT, SVM models were developed. The top two models were tested and reported.<br>10-fold CV was conducted	Accuracy (MLP = 0.898); R-squared (MLP = 0.6516, LR = 0.3214); RMSE (MLP = 0.2251, LR = 0.3887)	[48]
	Trauma patient data from the NTDB was extracted to develop an automated decision-making algorithm for triaging in mass casualty situations. Patients > 18 years, with blunt or penetrating trauma injuries were included.<br>The following exclusion criteria was used: (1) Data with missing vital signs or GCS values; (2) abnormal vital signs or GCS value; (3) Injury type grouped as burned; (4) vitals recorded in EMS	LR, RF, DNN models were developed using 5 input variables: age, HR, SBP, shock index (SI), SCS.<br>10-fold validation was implemented as a cross-validation method.	**AUROC** (LR = 0.844, RF = 0.882, DNN = 0.883); F1-score (LR = 0.673, RF = 0.783, DNN = 0.784); MMAE (LR = 0.387, RF = 0.297, DNN = 0.298).<br>DNN performed best out of all three models. An automated decision-support model provides a quick method of triaging patients during mass casualty incidents based on their degree of risk.	[49]
	A simulation-based study using NTDB and SweTrau data to develop models for triaging based on degree of risk. The inclusion criteria was any patient above 15 years of age. Missing values, unrealistic vitals (SBP > 300, RR > 67) and any uncertain readings (SBP = 0) were excluded	XGBoost model was compared with LR to assess its performance to appropriate triage trauma cases.<br>Model performances were assess using overt-and undertriaging (Overtriaging was defined as FP, and undertriaging was defined as FN).<br>Variables used to build the models were SBP, RR, GCS, and age	Using SweTrau data: **AUROC** (XGBoost = 0.725, LR = 0.725); **Overtriaging** (XGBoost = 0.322-0.319, LR = 0.323-0.321); **Undertriaging** (XGBoost = 0.314-0.324, LR = 0.312-0.321).<br>Using NTDB: **AUROC** (XGBoost = 0.611, LR = 0.614); **Overtriaging** (XGBoost = 0.463, LR = 0.468); **Undertriaging** (XGBoost = 0.406, LR = 0.395).<br>The over- and undertriaging rates for the two models were similar in performance – LR required a smaller training set to obtain a robust response	[50]

	Data obtained from the NTDB on trauma patients was used to develop a novel triage decision assisting model. Only blunt and penetrating trauma cases were included	LR (CAPSO) and NN (NN-CAPSO) were developed, and compare with other commonly used trauma scoring methods<br>Features used: GCS, age, PR, SBP, SpO_2	**AUROC** (NN-CAPSO = 0.921, CAPSO = 0.904, RTS = 0.851, NTS = 0.898, MGAP = 0.898, GAP = 0.897, TRIAGES = 0.903, TRISS = 0.934); **Accuracy** (NN-CAPSO = 0.578, CAPSO = 0.515, RTS = 0.873, NTS = 0.418, MGAP = 0.476, GAP = 0.406, TRIAGES = 0.380, TRISS = 0.594); **Sensitivity** (NN-CAPSO = 0.951, CAPSO = 0.960, RTS = 0.760, NTS = 0.938, MGAP = 0.952, GAP = 0.967, TRIAGES = 0.976, TRISS = 0.959); **Specificity** (NN-CAPSO = 0.559, CAPSO = 0.492, RTS = 0.879, NTS = 0.391, MGAP = 0.451, GAP =0.377, TRIAGES = 0.349, TRISS = 0.575).<br>NN-CAPSO outperformed the other trauma scores, and was able to more accurately classify patients to the right risk groups	[51]
	Adult trauma patients with “medium activation” presenting via helicopter to a level 1 trauma center from May 2007 to May 2009 were used.<br>(A “medium activation” trauma patient was defined as alert and hemodynamically stable on scene with either subnormal vital signs or accumulation of risk factors that may indicate a potentially serious injury; patients with burns as a major complaint, and patients aged < 18, and patients who arrived with the “highest activation” were excluded)	One thousand six hundred fifty three patients were included in the RF model.<br>Data was split into 70:30 for training and testing purposes.<br>The final model was externally validated using the same set of variables on a naive data set.<br>Eighty-three attributes relating to demographics and injury data such as mechanism of injury, incident details, triage accuracy, patient management characteristics (analgesia and crystalloid administration), bleeding status, pulse character, anatomic site of injury, type of injury at that anatomic site, range of vital signs as expressed by minimum and maximum values, prehospital fluid, medications, vitals (SBP, DBP, HR, SpO_2, RR), GCS collected as individual components	**Precision = PPV** = 0.34; **NPV** = 0.92; **Sensitivity** = 0.89; **Specificity** = 0.42.<br>In the testing set, there was an over triage rate of 66%, whereas using the RF, we decreased the over triage rate to 42% and 50% overall both better than our current practice	[52]
	Data on patients with traumatic hemorrhagic shock from the PLA General Hospital Emergency Rescue Database (PLAGH-ERD) and Medical Information Mart for Intensive Care III (MIMIC III) was used	XGBoost and LR models were developed. For the XGBoost models, the best hyperparameters were determined by grid search with 10-fold CV. For LR, Lasso method was used to prevent overfitting.<br>The dataset was split into 80:20 training and testing sets	Performance at 0.5 h: **AUROC** [LR = 0.875, XGBoost = 0.935].<br>The XGBoost model performance was significantly better than the logistic regression	[53]

	ICU patient stay data was collected from the Mayo Clinic Electronic Health Record for development of a decision support system	Trauma Triage Treatment and Training Decision Support (4TDS) system was built through a literature review, rapid prototyping, and design requirements review and combined features of two Decision Support Systems: Cooperative Communication System and Ambient Warning and Response Evaluation	Algorithms will be compared with actual clinical decisions in a 'silent test'.<br>HR, RR, SpO_2 and BP showed statistical significance in the high model performance	[54]
	Through the Michigan Trauma Quality Improvement Program (MTQIP) 22,069 trauma patients were used for model development。<br>Patients directly admitted or who arrived dead were excluded	Four classifiers were developed: (a) logistic regression, (b) random forest, (c) Boosted Tree (d) and support vector machines Three feature selection methods used to reduce the number of input variables to those most useful for the model: (a) all features, (b) generalized local learning, (c) recursive feature elimination; 19 variables were considered: age, gender, firearm injury, insurance, legal intervention, penetrating injury, fall injury, unintentional injury, central gunshot wound, field GCS, field SBP, field pulse, ED temperature, transport time < 15 min, transport time < 30 min, and evening arrival,	Boosted Tree: **AUROC** = 0.85.<br>The best feature selection and classifier combination was using all features with the boosted tree to accurately predict the need for a full trauma team activation using NEI-6 as the definition of major trauma.<br>The final model included age, gender, insurance, firearm injury, legal intervention, penetrating injury, fall injury, unintentional injury, central gunshot wound, field GCS, field SBP, field pulse, ED temperature, transport time < 15 min, transport time < 30 min, and evening arrival	[55]
	Pediatric Emergency Care Applied Research Network (PECARN) data between May 2007 and January 2010 was used	Various models were developed and compared: GLM, LDA, RF, SVM, SVMR, RPART, Naïve.<br>Data was split into 60:40 training testing sets.<br>SMOTE was used for balancing the datasets.<br>Nineteen clinical variables including emesis, dyspnea, GCS score of < 15, visible thoracic or abdominal trauma, seatbelt sign, abdominal distension, tenderness or rectal bleeding, peritoneal signs, absent bowel sounds, flank pain, pelvic pain or instability, sex, age, HR, and RR	Low-Risk model: **AUROC** (GLM = 0.96, RF = 0.99, SVM = 0.99, LDA = 0.90, SVMR = 0.8, PART = 0.75);<br>High-Risk model: **AUROC** (GLM = 0.96, RF = 0.99, SVM = 0.99, LDA = 0.96, SVMR = 0.99, RPART = 0.88);<br>All models except SVM had superior predictive power for identifying low-risk children compared with the naive rate. By both ROC and sensitivity, the fitted RF, SVMR, and SVM algorithms performed the best for identifying high-risk children	[56]

	All the trauma patients of over 16 years were included in the study。<br>Patients with incomplete data and victims who were dead on arrival were excluded of the study.<br>Missing age values were filled to the median value. The missing HR data were filled with mean. Missing data on the airway specificity of the injuries were filled with mode	SVM, KNN, Bagging, Adaboost, NN algorithms were developed.<br>Data was split into 70:30 training testing sets.<br>The most-fitted variables were GCS score, base deficit, and DBP In all the ranked sets, the index after resuscitation included GCS, HCT, DBP, Base excess, pH, PO2 and HCO3 with high ratings	**Accuracy** (SVM = 0.9924, KNN = 0.694, Bagging = 0.9967, Adaboost = 0.7581, NN = 0.516); **Sensitivity** (SVM= 0.694, KNN = 0.7427, Bagging = 0.9804, Adaboost = 0.721, NN = 0.5769); **F-Measure** (SVM = 0.764, KNN = 0.7056, Bagging = 0.9896, Adaboost = 0.722, NN = 0.6045).<br>Bagging algorithm has the best result among the algorithms used, followed by the SVM method to predict illness of patients	[57]
	Gunshot wound patient data were collected from ACS-TQIP	A Dirichlet-deep NN (field artificial intelligence triage) was developed.<br>Data was split into 80:20 training testing sets	Field artificial intelligence triage outperformed all other tested methods ML algorithms, including logistic regression, k-nearest neighbors, support vector machines, random forests (RFs), and conventional deep NNs (DNN-CE)) on all prediction tasks	[58]
	Subjects for the present study were healthy, non-smoking, normotensive males or non-pregnant females, with ages ranging from 18 years to 55 years	LBNP was conducted on subjects to simulate hemorrhage in humans. Continuous waveform data were collected at 500 Hz using WinDaq data acquisition software.<br>The test subject data were termed test data, and data from all other subjects were termed learning data, with no mixing between the two sets.<br>The CRI models were built using Finometer waveform data from 183 LBNP subjects and were tested on the 184th. This process was repeated 184 times	This study identified several waveform features that can be used in the CRI algorithm and to develop a human model of severe acute blood loss	[59]
	Data from soldiers who were evacuated from Iraq/Afghanistan after sustaining injuries to one or more extremities to develop a decision supporting tool to guide the timing of wound closures.<br>Participants were included if they had at least one extremity wound > 75 cm^2 with negative pressure wound therapy	RF (all features), RF (10 most important features), BBN (12 features), and LR (using LASSO and requiring 8 features) models were developed.<br>Features (Biomarkers) that were statistically significant to wound outcome - Serum IL7, Effluent IL4, Serum IL1a, Serum MCP-1, Effluent IL-6, Genitourinary trauma and the transfusion requirement.<br>10-fold CV was implemented for cross validation and the data was split into 90:10 training testing sets	**AUROC** [RF (all) = 0.72, RF (10) = 0.79, BBN = 0.74, LR = 0.62]<br>RF (using 10 features) performed best and was associated with the highest net benefit. Use of this model would improve clinical outcomes as it would reduce any unnecessary surgical procedures. The model presents ability to predict accurately in a generalizable setting (civilians and military)	[60]

	Trauma patients triaged based on the recommendations of the French Society of Emergency Medicine (SFMU).<br>Patients who experienced a cardiac arrest before arrival; Patients with missing data on the first HR. Other missing vital signs were manageable as they could be representative of patient severity	Developing a DT model to provide early clinical decision-making for identification of care based on injury severity.<br>The model is compared with the Mechanism, GCS, age and arterial pressure (MGAP) score.<br>10-fold cross-validation were employed	**AUROC** [MGAP (threshold 22) = 0.59, DT = 0.82]; **Sensitivity =** [MGAP = 0.26, DT = 0.94]; **Specificity** [MGAP = 0.91, DT = 0.48].<br>DT model with a threshold of 0.345 resulted in a higher sensitivity and lower specificity than a threshold of 0.634	[61]
	Three hundred and thirty-eight cases (minor injury,<br>114 cases; moderate injury, 82 cases; serious injury, 74 cases; and critical injury and death, 68 cases) complete with the available data to form the test set	The RF, XGBoost, and NB models and DNN model were trained with the same training set and then tested with the same real samples.<br>Synthetic samples were used as the training set with 22 features, real samples as the test set. A war trauma severity scoring algorithm (WTSS) was used for data augmentation	Performance for WTSS method/MA method: **Accuracy** (WTSS-DNN = 0.8443/0.7124 RF = 0.8146/0.6939, XGBoost =0.8208/ 0.6886, NB = 0.6408/0.5386); **Precision = PPV** (WTSS-DNN = 0.9007/0.7266, RF = 0.8506/0.7021, XGBoost =0.8200/0.6912, NB = 0.6860/0.5520); **Sensitivity =** (WTSS-DNN = 0.8844/0.7081 RF = 0.8301/0.7047, XGBoost = 0.8507/0.7007, NB = 0.0.6356/0.5341); **F-Measure** (WTSS-DNN = 0.8925/0.7034, RF = 0.0.8402/0.7034, XGBoost =0.8351/0.6959, NB = 0.6598/0.5429).WTSS algorithm outperformed the manual evaluation (MA) method for each of the models. WTSS-DNN was the best performing ML model	[62]

T	A retrospective study of 1371 adult patients with external trauma admitted to the Emergency Department to develop and compare ML models that predict whether blood transfusion is needed after trauma	Statistical logistic regression (LR), machine learning decision tree algorithm [classification and regression tree (CART) and XGBoost] for prediction of RBC demand.<br>The numerical variables were extracted directly, including Non-invasive vital sign parameters, and invasive laboratory test results, trauma severity classification (first level, second level and third level), endotracheal intubation and vasoactive drugs and information related to blood transfusion in the database	Non-invasive parameters: **AUROC** (LR = 0.72, CART = 0.69, XGBoost = 0.71); **Accuracy** (LR = 0.55, CART = 0.48, XGBoost = 0.75); **Sensitivity =** (LR = 0.86, CART = 0.89, XGBoost = 0.66); Specificity (LR = 0.77, CART = 0.50, XGBoost = 0.42).<br>The trauma location and shock index are important prediction parameters.<br>For all the prediction parameters: **AUROC** (LR = 0.80, CART = 0.82, XGBoost = 0.94); **Accuracy** (LR = 0.72, CART = 0.89, XGBoost = 0.83); **Sensitivity =** (LR = 0.80, CART = 0.69, XGBoost = 0.94); Specificity (LR = 0.70, CART = 0.92, XGBoost = 0.82).<br>Haematocrit (Hct) is an important prediction parameter.<br>For non-invasive parameters, LR was best, and for all parameters, XGBoost was best	[63]
	Retrospective Data from patients who sustained traumatic injuries were used to develop ML models to predict need for transfusion	LR, SVM, NN, RF models were used. Important variables used are age, gender, mechanism of injury, involvement in explosion, vital signs.<br>70:30 training testing split.	**AUROC** (LR = 0.9637, RF = 0.984, SVM = 0.9677, NN = 0.9489); **Accuracy** (LR = 0.9087, RF = 0.9598, SVM = 0.9219, NN= 0.9042); **Precision** (LR = 0.8991, RF = 0.9537, SVM= 0.9065, NN = 0.8879); **Specificity** (LR = 0.8871, RF = 0.949, SVM =0.8941, NN = 0.8724); (LR = 0.9287, RF = 0.9698, SVM = 0.9476, NN = 0.9355); **NPV** (LR = 0.9199, RF = 0.9667, SVM = 0.9403, NN = 0.9237); **F1-Score** (LR = 0.9137, RF = 0.9617, SVM = 0.9266, NN = 0.9102).<br>All models displayed high performance metrics; RF outperformed the other models	[64]
	An observational study of patients meeting the trauma center activation criteria at a level 1 trauma center were observed. Subjects were included on whom the trauma team was activated. Patients < 18 years, prisoners, and pregnant women were excluded form the study. The CRI device is worn by the subject to allow for a wide time analysis	The CRI device uses a ML algorithm to analyse and evaluate the subject's arterial waveform.<br>Subjects who were experiencing hemorrhage had significantly higher length of stay, ISS, injury care length of stay.<br>LR analyses were built to predict hemorrhage between CRI < 0.70 and SBP < 110	**AUROC** (SBP = 0.62, CRI = 0.83); **Sensitivity** (SBP = 0.25, CRI = 0.83); **Specificity** (SBP = 0.94, CRI = 0.60); **PPV** (SBP = 0.60, CRI = 0.43); **NPV** (SBP = 0.77, CRI = 0.91).<br>CRI demonstrated a better performance at being able to predict posttraumatic hemorrhaging than SBP	[65]

	Retrospective study using the UK Joint Theatre Trauma Registry to develop a decision rule for on-hospital arrival patients suffering battlefield trauma. Subjects were included if vital sign data was available at some point during their resuscitation. Children < 16 years of age were excluded	Three categories of variables were used to develop the binary LR model (MASH): (1) Clinical variables – PR, SBP, GCS, RR, and temperature; (2) Injury patterns - type of injury); (3) Pre-Hospital data – tourniquet, use of hemostatic agents	Performance for a score of 3: **AUROC** = 0.93; **Sensitivity** = 0.827; **Specificity** = 0.888; **PPV** = 0.35; **NPV** = 0.99.<br>MASH score has a higher sensitivity and specificity than other previous military prediction tools, with no reliance on lab investigative information	[66]
	Data from all trauma patients were used to develop a model that can accurately predict massive transfusion cases	A predictive model using the least absolute shrinkage and LASSO regression.<br>80:20 training and testing split.<br>10-fold CV.<br>LASSO was used for data balancing, preventing biasing and overfitting	**AUROC** = 0.96; **Accuracy** = 0.956	[67]
	Data using the PROMMTT dataset was used on patients who were transfused at least one unit of pRBCs upon hospital arrival	Validation of the model using the different definitions of MT (Mt 1-6). These results were compared with other commonly used MTP prediction models like ABC and TASH.<br>Variables collected include patient demographics, SBP, HR, ISS, mechanism of injury, transfusion details, and survival	**AUROC** (Mt1 = 0.694, Mt2 = 0.698, Mt3= 0.711, Mt4 = 0.700, Mt5 = 0.696, Mt6 = 0.694, ABC = 0.620, TASH = 0.527).<br>The app models using all 6 definitions of MT all outperformed the other MT prediction models	[68]
	Trauma patient data collected during transportation to hospital was used to train and analyse the various models for transfusion prediction.<br>Patients with continuous vital sign data during pre-hospital transfer was included. Patients who died within 15 min of admission was excluded	Bleeding Risk Index (BRI) was developed, and compared with RTS, SI, and ABC.<br>The model was compared on its ability to predict Critical Administration Threshold (CAT), and Massive Transfusion (MT).<br>CAT was defined as ⩾ 3 units of pRBC in the first hour after admission.<br>MT was defined as ⩾10 units of pRBC in the first 24 h	Performance for CAT prediction/MT prediction: **AUROC** (BRI = 0.91/0.92, ABC = 0.77/0.8, SI = 0.85/0.83, RTS = 0.78/0.78); **PPV** (BRI = 0.31/0.16, ABC = 0.25/0.12, SI = 0.32/0.14, RTS = 0.18/0.08); **NPV** (BRI = 0.98/0.99, ABC = 0.97/0.99, SI = 0.98/0.99, RTS = 0.97/0.99).<br>BRI outperforms the other transfusion scores	[69]
	All patients diagnosed as multiple trauma and adult patients with age of 18 years and older were included.<br>Pregnant woman, patients diagnosed with traumatic brain injury, serious cardiovascular and cerebrovascular diseases or serious hematologic disorders were excluded	A decision tree model was developed to predict the occurrence of MT.<br>The dataset was split into 50:50 training testing sets.<br>Shock index, injury severity score, international normalized ratio, and pelvis fracture were the most significant risk factors of MT.<br>In the training model, 12 variables were used as input variables.<br>The INR, SBP, ISS, and injury type remained in the model	**AUROC** = 0.86; **Accuracy** = 0.89, **Sensitivity** = 0.80, **Specificity** = 0.90.<br>A MT prediction model is established using the decision tree algorithm and evidently has a good predictive performance	[70]

	The charts for all trauma injury patients admitted into the ER were used. The records of patient records identified patients that had received a transfusion of at least one type of blood product	A total of 1016 patient records are used to train and test a backpropagation NN for predicting the transfusion requirements of these patients during the first 2, 2-6, and 6-24 h, and for total transfusions. Both one and two hidden layer ANNs trained using the backpropagation learning algorithm are implemented. Multiple multinomial LR model was used for total pRBC.<br>The data was split into 70% training: 30% testing dataset.<br>Nine variables were identified: age, sex, race, etiology of trauma, type of trauma, if safety equipment was in use, the GCS for the patient, RR and SBP	**Accuracy** (ANN for RBC/FFP/platelet = 0.6778/0.8264/0.705, LR for pRBC = 0.5105); **Sensitivity** (ANN for RBC/FFP/platelet = 0.9247/0.6923/0.717, LR for pRBC = 0.7035); **Specificity** (ANN for RBC/FFP/platelet = 0.431/0.8475/0.7035, LR for pRBC = 0.0377).<br>ANNs can accurately predict most ER patient transfusion requirements while only using information available at the time of entry into the ER	[71]
	Children (<18 years) who sustained a blunt solid organ injury between 2009-2018 were included	For the MT model, a training set of 37 was used and a validation set of 440 was used. For the failure of NOM model, a training set of 47 and a validation set of 430 was used. For the mortality model, a training set of 30 and a validation set of 447 was used. Lastly, for the successful NOM without intervention model, a training set of 66 and validation set of 411 was used.<br>demographics (gender, age, weight), GCS scores, clinical values [vital signs, shock index-pediatric adjusted (SIPA), organ injured, and blood products received], laboratory results [hemoglobin, base deficit, INR, lactate, thromboelastography (TEG)], and imaging findings [focused assessment with sonography in trauma (FAST) and grade of injury on computed tomography scan] from prehospital to ED settings	4 h model predictive performance: **AUROC** (MT= 0.9, Failure of NOM = 0.88, mortality = 0.96, successful NOM = 0.89); **Accuracy** (MT = 0.905, Failure of NOM = 0.838, mortality = 0.919, successful NOM = 0.903); **Sensitivity** (MT = 0.889, Failure of NOM = 0.917, mortality = 0.100, successful NOM = 0.904); **Specificity** (MT = 0.905, failure of NOM = 0.835, mortality = 0.918, successful NOM = 0.882);<br>The four-hour models outperformed the 24 models for all outcomes	[72]
	Trauma patients who received at least one unit of RBCs and/or low-titer group O whole blood between January 1, 2015, and December 31, 2017 from the University of Pittsburgh Medical Center (UPMC) records.<br>Patients who received at least one unit of RBCs are included.<br>Patients did not have complete clinical and demographic data available were excluded	A recursive partitioning algorithm was used to generate two decision trees for prediction of massive transfusion: (1) using parameters easily available during ED admission (sex, SBPHR, GCS, RR, FAST scan, mechanism of injury (penetrating vs. blunt), presence of an open or dislocated femur fracture, and presence of a clinically unstable pelvic fracture); (2) using same parameters as (1) + laboratory variables (hemoglobin, INR, pH, and base deficit).<br>Data was split 2:1 training and validation sets.<br>10-fold CV was implemented	**Accuracy** (MtPitt = 0.769, MtPitt+Labs = 0.879); **Precision** (MtPitt = 0.228, MtPitt+Labs = 0.371); **NPV** (MtPitt = 0.986, MtPitt+Labs = 0.988); **Sensitivity** (MtPitt = 0.867, MtPitt+Labs = 0.867); **Specificity** (MtPitt = 0.761, MtPitt+Labs = 0.88).<br>MTPitt + Labs decision tree showed the highest sensitivity, balanced accuracy, and Youden J index, compared to the MTPitt decision tree and the Assessment of Blood Consumption (ABC) and Trauma Associated Severe Hemorrhage (TASH) scores	[73]

HD	Twenty-four volunteers were exposed to an LBNP protocol (data from 21 was used).<br>Patients with linear increases in HR were included. Pregnant subjects were excluded from the study	Subjects wore a SenseWear Pro2 Armband that measured Heat flux (HF), HR, skin temperature, galvanic skin response, EKG.<br>A ML algorithm was used to analyse the physiological waveform data to detect LBNP at different stages of the exercises (1-5)	**Accuracy** (exercise 1 = 0.938, exercise 2 = 0.955, exercise 3 = 0.960, exercise 4 = 0.946, exercise 5 = 0.946); **Precision** (exercise 1= 0.926, exercise 2 = 0.955, exercise 3 = 0.942, exercise 4 = 0.930, exercise 5 = 0.936); **Sensitivity** (exercise 1= 0.952, exercise 2 = 0.971, exercise 3 = 0.981, exercise 4 = 0.964, exercise 5 = 0.955); **Specificity** (exercise 1= 0.924, exercise 2 = 0.938, exercise 3= 0.940, exercise 4 = 0.929, exercise 5 = 0.938).<br>This method provides a non-invasive way of measuring stroke volume (SV)	[74]
	190 healthy participants were placed in a Lower Body Negative Pressure device (LBNP) to simulate and observe the early stages of hemorrhage.<br>Continuous non-invasively measure hemodynamic signals were measured from the subjects for developing the ML algorithm	A linear and non-linear density model (in real time) was implemented into an image-based robot navigation system to train and predict blood volume loss	**Accuracy** = 0.965; **R-squared** = 0.7921	[75]
	24,996 non-contrast head CT scan data from adult trauma patients were used to develop an ICH detection algorithm	A Natural Language Processing tool was used to predict hemorrhaging	**Precision = PPV** = 73; **NPV** = 94; **Sensitivity** = 73; **Specificity** = 94	[76]
	Data taken from 627 trauma injured patients during transport to ED. The aim of the study was to identify a hypovolemic state in trauma patients. Patient records with each of the vital signs present during the 5 – 7 min interval of pre-hospital arrival were included. Patients who received blood but did not meet the document injury criteria were excluded	Linear classifiers were implemented to discriminate between two classes - control and hemorrhage, which are then combined into an ensemble classifier. The classifier was trained using 5 vital sign variables – HR, RR (RR), DBP, and SBP	**AUROC** = 0.76; **Sensitivity** = 0.69; **Specificity** = 0.68.<br>HR and SBP seem to be the common best features extracted for developing the model.<br>Classification degrades slowly as variables are dropped in the ensemble classifier, highlighting its robustness. It outperforms other linear classifiers	[77]
	CT scan data from 12 pelvic trauma patients (each scan having 30 – 70 images) were taken to develop an automatic hemorrhage detection and segmentation model	An SVM model that uses pelvic anatomical information to segment hemorrhage accurately was developed using the CT scans of pelvic trauma patients. The data was validated using a 10-fold CV mechanism.	**Accuracy** = 0.9428.<br>Very little missegmentation of the scans, which allows faster diagnostic decision (as opposed to manual segmentation and analysis)	[78]

	Patient vital sign records from 492 trauma casualties during the 5 – 7 min interval of pre-hospital arrival was used in developing a model for the detection of major hemorrhaging. Patients who received blood but did not meet the document injury criteria were excluded. Records with missing values were also excluded.	Linear (Linear discriminant function), and non-Linear (ANN and SVM) were implemented. The ANN model was a three layer, with two hidden node and one output node, trained with a conjugate gradient algorithm.<br>ECG, PPG, monitor calculated variables (HR, RR, SpO_2, SBP and DBP).<br>A split of 60%:40% was done for training and test data resp, through 100 simulation tests	The best two features are SBP and HR. The use of non-linear classifiers did not improve the discrimination, as the linear classifier had the best performance.	[79]
	Retrospective data from the French Trauma registry, using all patients hospitalized for severe trauma. Only patients with Hemorrhagic Shock (HS), and Traumatic Brain Injury (TBI) were included. Patients transferred or < 15 years were excluded from the study	Poisson regression model was developed.<br>HS was based on transfusion ≥ 4 RBC packs, while TBI was based on an AIS head score > 1.<br>The following variables were used: epidemiological data, GCS, SBP, DBP, HR, haemoglobin.<br>Amount of RBC packs, platelets and fresh frozen plasma transfused, transfusion timing, and coagulation tests results were additional taken for HS. For TBH intracranial hypertension treatment, and treatment of secondary cerebral insults were also collected	**AUROC** (HR = 0.92, TBI = 0.97).<br>The Poisson regression model provided minimal recommendations, which would reduce the 7-day mortality rate for TBI and HS cases	[80]
	898 trauma-injured patients during transport by medical helicopter from the scene of injury to the Level I unit were collected.<br>patients did not have any vital-sign data collected during transport, patients with ambiguous hemorrhagic condition, and unreliable vital-sign data based on the data reliability algorithm were excluded	each ensemble classifier was trained and tested through 100 trials, each using 50% of the data for training and the remaining 50% for testing.<br>vital signs: HR, RR (RR), arterial blood SpO_2, and systolic and DBPs (BPs; SBP and DBP, respectively)	**AUROC** (Ensemble = 0.85, SBP < 110 mmHg = 0.71); **Sensitivity** (Ensemble = 0.85, SBP < 110 mmHg = 0.47); **Specificity** (Ensemble = 0.73, SBP < 110 mmHg = 0.87)	[81]
	65 studies were routinely acquired with 64 section or higher MDCT scanners in the trauma bay in either the late arterial or portal venous phase of enhancement	A multi-scale attentional network (MSAN) model was developed	The proposed MSAN substantially improves the segmentation accuracy by more than 7% compared with prior arts	[82]

	373 urgent NCCT scans performed at a single academic medical center were used in the development of the ICH detection model	A commercially developed convolutional neural network algorithm developed by Aidoc for ICH detection was utilized. Algorithm was trained and tested on CT scans from 9 different centers and 17 different scanners.	Aidoc for all cases (**Sensitivity** = 0.887; **specificity** = 0.942; **PPV** = 0.737; **NPV** = 0.977; **accuracy** = 0.934). Aidoc for emergency cases (**Sensitivity** = 0.863; **specificity** = 0.970; **PPV** = 0.587; **NPV** = 0.993; **accuracy** = 0.965)	[83]
	Datasets of CT scans collected from 9 different sites. Aim of the study was to assess the impact of implementing a commercial AI software to analyze and detect any intracranial hemorrhage (ICH) – unclear whether trauma patients or not	A commercially developed deep learning triage software developed by Aidoc was utilized	Aidoc (Precision = 0.859, NPV = 0.963, Sensitivity = 0.884, Specificity = 0.961	[84]
	Data from Trauma patients with penetrating injury were collected for the BRI development Patients dying within 15 min of trauma center arrival and those younger than 18 years were excluded	Bleeding Risk Index (BRI) based on features from pulse oximetry and electrocardiography waveforms and BP (BP) trends was calculated to predict patients who were undergoing REBOA, RT, and predict mortality outcome.<br>Forty features derived from photoplethysmography (PPG) and electrocardiography (ECG) waveforms captured at 250 Hz and continuously collected including pulse oximetry (SpO_2) numeric values and SBP trends captured at 1 Hz	**AUROC** (BRI = 0.93, SI = 0.72); **Precision** (BRI = 0.08); **NPV** (BRI = 0.99); **Sensitivity** (BRI = 0.95); **Specificity** (BRI = 0.85)	[85]
C	Fifty-four trauma patients with rapid-TEG samples collection within one hour of surgery, who had not received any prior blood products were used to predict Trauma induced coagulopathy (TIC). Trauma patients with pre-existing renal disease, coagulopathic disorder or using anticoagulant or antiplatelet drugs were excluded from the cohort	A Decision Tree was developed. Rules to develop the tree structure: (1) ACT < 103, (2) ACT < 103 and MA < 72.55 = control, (3) ACT < 103 and MA ⩾ 72.55 = ESRD, (4) ACT ⩾ 103 and MA < 60.8 = ACOT, (5) ACT ⩾ 103 and MA ⩾ 60.8 = ESRD	**Accuracy** = 0.934.<br>End stage renal disease (ESRD) and TIC demonstrated distinct TEG patterns. The DT Classifier forms the basis for a clinical decision support software for viscoelastic hemostatic assays (VHA)	[86]

	A total of 818 patients from Emergency Rescue Database were included as derivation cohort, and a total of 567 patients further collected from ED were included in the study as validation cohort	Random forest and traditional logistic regression were deployed for prediction modeling of ATC (international normalized ratio (INR) values >1.5 upon admission to the ED	**AUROC** (RF = 0.810, LR = 0.849); **Accuracy** (RF = 0.94, LR = 0.935); **Precision** (RF = 0.933, LR = 0.931); **F-measure** (RF = 0.934, LR = 0.92); **Sensitivity =** (RF = 0.94, LR = 0.935).<br>The accuracy, precision, F1 score, and Sensitivity of the RF model was higher but yielded lower AUROC score.<br>for predicting ATC in the trauma patients.<br>Compared to the logistic regression model, the RF model showed better accuracy	[87]
	Trauma patients admitted to emergency department are used as the dataset (after patients not meeting inclusion criteria are excluded).<br>The inclusion criteria is based on the following: (1) < 30 d from injury to admission; (2) age ≥ 18 years old; (3) length of stay > 3 d; Patients diagnosed with VTE upon admission are excluded.<br>Any missing value is filled through mean values	The following models are developed and compared with the Caprini score predictor: RF model based on DT for prediction modelling (with different feature screening ML methods like Least Absolute Shrinkage and selection operator (LASSO), Ridge, ElasticNet, LR, Mutual Information Entropy (MIE) regression).<br>Two different model sets are implemented: (1) using the patient data, (2) patient data + Caprini score.<br>A 10-fold validation is implemented for cross validation	Model set (1): **AUROC** (LASSO+RF = 0.759, Ridge + RF = 0.760, ElasticNet + RF = 0.763, LR + RF = 0.769, MIE + RF = 0.759, Caprini = 0.773); **Accuracy** (LASSO+RF = 0.636, Ridge + RF = 0.620, ElasticNet + RF = 0.635, LR + RF = 0.619, MIE+RF = 0.642, Caprini = 0.750); **Precision** (LASSO+RF = 0.332, Ridge + RF = 0.324, ElasticNet + RF = 0.331, LR + RF = 0.329, MIE + RF = 0.334, Caprini = 0.444); **TPR** (LASSO+RF = 0.692, Ridge + RF = 0.707, ElasticNet + RF = 0.685, LR + RF = 0.718, MIE + RF = 0.684, Caprini = 0.667); **FPR** (LASSO+RF = 0.320, Ridge + RF = 0.340, ElasticNet + RF = 0.310, LR + RF = 0.330, MIE + RF = 0.310, Caprini = 0.227).<br>Caprini score predictor, RF model based on DT for prediction modelling (with different feature screening ML methods like Least Absolute Shrinkage and selection operator (LASSO), Ridge, ElasticNet, LR, Mutual Information Entropy (MIE) regression)	[88]

	The development cohort comprised data from consecutive patients enrolled in the ACIT study between January 2007 and October 2011 at RLH. The model was validated in new patients enrolled into the ACIT study.<br>Adult patients (>15 years) presenting directly to participating Major Trauma Centers, who meet local criteria for trauma team activation, are included into the study. Patients are excluded if they meet any of the following: (1) no consent given, (2) take anticoagulants medication, (3) have moderate or severe liver disease, (4) have a bleeding diathesis	BN prediction model was developed using data from 600 patients recruited into the Activation of Coagulation and Inflammation in Trauma (ACIT) study.<br>Performance was tested using 10-fold cross-validation.<br>HR, SBP, temperature, hemothorax, FAST result, Unstable pelvic fracture, Long bone fracture, GCS, Lactate, Base deficit, pH, Mechanism of Injury, Energy	BN Model performance with internal validation/external validation: **AUROC** (0.96/0.93).<br>This model showcases a method of predicting an individual's risk of TIC using clinical information<br>This information may be used to support early and rational decisions on the use of damage control interventions and guide rapid and efficient activation of damage control resuscitation protocols, which in turn, may prevent an established coagulopathy and lead to improved outcomes	[89]

C coagulopathy, *HD* hemorrhage detection, *IS* injury severity, *RA* risk assessment, *T* transfusion, *TO* trauma outcome, *ACS-TQIP* American College of Surgeons Trauma Quality Improvement Program, *AI* Artificial intelligence, *AIS* Abbreviated Injury Scale, *ANN* Artificial Neural Network, *ASCOT* a severity characterization of trauma, *AUROC* area under the receiver operating curve, *BBN* bayesian belief network, *BD* base deficit, *BE* backward elimination, *BN* bayesian network, *BP* blood pressure, *BRI* bleeding risk index, *CART* classification and regression tree, *CRI* critical reserve index, *CT* computed tomography, *CV* cross validation, *DBP* diastolic blood pressure, *DNN* deep NN, *DT* decision tree, *ED* emergency department, *EKG/ECG* electrocardiogram, *ER* emergency room, *ER* evidential reasoning, *ESRD* end-stage renal disease, *FAST* focused assessment with Sonography for Trauma, *FFP* fresh frozen plasma, *FGF* fibroblast growth factor, *FIS* fuzzy inference system, *GCS* Glasgow Coma Score, *GNB* Gaussian Naïve Bayes Classifier, *HR* heart rate, *ICH* intracranial hemorrhage, *ICU* Intensive Care Unit, *IL* interleukin, *INR* international normalized ratio, *ISS* injury severity score, *KNN* k-nearest neighbor algorithm, *LASSO* least absolute shrinkage and selection operator, *LDA* linear discriminant analysis, *LBNP* low body negative pressure, *MAP* mean arterial pressure, *MDCT* multidetector CT, *MGAP* Mechanism, Glasgow coma score, Age and Arterial Pressure, *MLP* multi-layer perceptron model, *MLR* multivariate logistic regression, *MT* massive transfusion, *NCCT* non-contrast head CT, *NLP* natural language processing, *NPV* negative predictive value, *NTDB* national trauma data bank, *OCT* optimal classification trees, *PART* partitioning decision tree, *PI* permutation imputation, *PPG* photoplethysmosgram, *PPM* personal predictive monitoring, *PPV* positive predictive value, *PR* pulse rate, *PROMMTT* Prospective, Observational, Multicenter, Major Trauma Transfusion, *R2* correlation coefficient, *RAM* risk assessment model, *RBC* red blood cell, *RBFN* radial-basis function network, *RF* random forest, *RLH* Royal London Hospital, *RR* respiration rate, *RSNNS* stuttgart neural network simulator, *RTS* revised trauma score, *SBP* systolic blood pressure, *SCS* simplified consciousness score, *SI* shock index, *SMOTE* synthetic minority over-sampling technique, SpO_2 oxygen saturation, *SVM* support vector machine, *TARN* trauma Audit and Research Network, *TBI* traumatic brain injury, *TIC* trauma induced coagulopathy, *TEG* thromboelastography, *TOP* trauma outcome predictor, *TSM* trauma severity model, *UKTARN* United Kingdom Trauma Audit Research Network database, *UKTRISS* United Kingdom Trauma and Injury Severity Score, *VTE* venous thromboembolism, *WBC* white blood cell, *WVSM* wireless vital signs monitor

References

1. Ahmed FS, Ali L, Joseph BA, Ikram A, Ul Mustafa R, Bukhari SAC. A statistically rigorous deep neural network approach to predict mortality in trauma patients admitted to the intensive care unit. J Trauma Acute Care Surg. 2020;89(4):736-42.

2. Almaghrabi F, Xu DL, Yang JB. An application of the evidential reasoning rule to predict outcomes following traumatic injuries. In: Li Z, Yuan C, Liu J, Kerre EE, editos. Developments of Artificial Intelligence Technologies in Computation and Robotics: Proceedings of the 14th International FLINS Conference (FLINS 2020). Cologne, Germany: World Scientific; 2020. p. 849-56.

3. Almaghrabi F, Xu DL, Yang JB. An evidential reasoning rule based feature selection for improving trauma outcome prediction. Appl Soft Comput. 2021;103:107112.

4. Becalick DC, Coats TJ. Comparison of artificial intelligence techniques with UKTRISS for estimating probability of survival after trauma. UK Trauma and Injury Severity Score. J Trauma. 2001;51(1):123-33.

5. Cardosi JD, Shen H, Groner JI, Armstrong M, Xiang H. Machine learning for outcome predictions of patients with trauma during emergency department care. BMJ Health Care Inform. 2021;28(1):e100407.

6. Chesney T, Penny K, Oakley P, Davies S, Chesney D, Maffulli N, et al. Data mining medical information: Should artificial neural networks be used to analyse trauma audit data? IJHISI. 2006;1(2):51-64. Available from: https://ideas.repec.org/a/igg/jhisi0/v1y2006i2p51-64.html

7. Christie SA, Conroy AS, Callcut RA, Hubbard AE, Cohen MJ. Dynamic multi-outcome prediction after injury: applying adaptive machine learning for precision medicine in trauma. PLoS One. 2019;14(4):e0213836.

8. Demšar J, Zupan B, Aoki N, Wall MJ, Granchi TH, Robert Beck J. Feature mining and predictive model construction from severe trauma patient's data. Int J Med Inform. 2001;63(1-2):41-50.

9. El Hechi M, Gebran A, Bouardi HT, Maurer LR, El Moheb M, Zhuo D, et al. Validation of the artificial intelligence-based trauma outcomes predictor (TOP) in patients 65 years and older. Surgery. 2022;171(6):1687-94.

10. DiRusso SM, Sullivan T, Holly C, Cuff SN, Savino J. An artificial neural network as a model for prediction of survival in trauma patients: validation for a regional trauma area. J Trauma. 2000;49(2):212-20.

11. Gorczyca MT, Toscano NC, Cheng JD. The trauma severity model: an ensemble machine learning approach to risk prediction. Comput Biol Med. 2019;108:9-19.

12. Hunter A, Kennedy L, Henry J, Ferguson I. Application of neural networks and sensitivity analysis to improved prediction of trauma survival. Comput Methods Programs Biomed. 2000;62(1):11-9.

13. Izenberg SD, Williams MD, Luterman A. Prediction of trauma mortality using a neural network. Am Surg. 1997;63(3):275-81.

14. Kilic YA, Konan A, Yorganci K, Sayek I. A novel fuzzy-logic inference system for predicting trauma-related mortality: emphasis on the impact of response to resuscitation. Eur J Trauma Emerg Surg. 2010;36(6):543-50.
15. Kim D, You S, So S, Lee J, Yook S, Jang DP, et al. A data-driven artificial intelligence model for remote triage in the prehospital environment. PLoS One. 2018;13(10):e0206006.
16. Kong GL, Xu DL, Yang JB, Yin XF, Wang TB, Jiang BG, et al. Belief rule-based inference for predicting trauma outcome. Knowledge-Based Systems. 2016;95:35-44.
17. Koukouvinos C, Parpoula C. Development of a model for trauma outcome prediction: a real-data comparison of artificial neural networks, logistic regression and data mining techniques. Int J Biomed Eng Technol. 2012;10(1):84-99.
18. Kuo PJ, Wu SC, Chien PC, Rau CS, Chen YC, Hsieh HY, et al. Derivation and validation of different machine-learning models in mortality prediction of trauma in motorcycle riders: a cross-sectional retrospective study in southern taiwan. BMJ Open. 2018;8(1):e018252.
19. Lee KC, Lin TC, Chiang HF, Horng GJ, Hsu CC, Wu NC, et al. Predicting outcomes after trauma: prognostic model development based on admission features through machine learning. Medicine (Baltimore). 2021;100(49):e27753.
20. Maurer LR, Bertsimas D, Bouardi HT, El Hechi M, El Moheb M, Giannoutsou K, et al. Trauma outcome predictor: an artificial intelligence interactive smartphone tool to predict outcomes in trauma patients. J Trauma Acute Care Surg. 2021;91(1):93-9.
21. Pearl A, Caspi R, Bar-Or D. Artificial neural network versus subjective scoring in predicting mortality in trauma patients. Stud Health Technol Inform. 2006;124:1019-24.
22. Rutledge R. Injury severity and probability of survival assessment in trauma patients using a predictive hierarchical network model derived from ICD-9 codes. J Trauma. 1995;38(4):590-7.
23. Rutledge R, Osler T, Emery S, Kromhout-Schiro S. The end of the Injury Severity Score (ISS) and the Trauma and Injury Severity Score (TRISS): ICISS, an International Classification of Diseases, ninth revision-based prediction tool, outperforms both ISS and TRISS as predictors of trauma patient survival, hospital charges, and hospital length of stay. J Trauma. 1998;44(1):41-9.
24. Wolfe R, McKenzie DP, Black J, Simpson P, Gabbe BJ, Cameron PA. Models developed by three techniques did not achieve acceptable prediction of binary trauma outcomes. J Clin Epidemiol. 2006;59(1):26-35.
25. Pearl A, Bar-Or R, Bar-Or D. An artificial neural network derived trauma outcome prediction score as an aid to triage for non-clinicians. Stud Health Technol Inform. 2008;136:253-8.
26. Pearl A, Bar-Or D. Using artificial neural networks to predict potential complications during trauma patients' hospitalization period. Stud Health Technol Inform. 2009;150:610-4.

27. Stojadinovic A, Eberhardt J, Brown TS, Hawksworth JS, Gage F, Tadaki DK, et al. Development of a Bayesian model to estimate health care outcomes in the severely wounded. J Multidiscip Healthc. 2010;3:125-35.
28. Schetinin V, Jakaite L, Jakaitis J, Krzanowski W. Bayesian decision trees for predicting survival of patients: a study on the US National Trauma Data Bank. Comput Methods Programs Biomed. 2013;111(3):602-12.
29. Zhang Y, Daigle B, Ferrigno L, Cohen M, Petzold L. Data-driven mortality prediction for trauma patients. Proc Annual Conf on Neurl Inf Proc Syst. Montreal, Canada; 2014. https://cse.cs.ucsb.edu/sites/cse.cs.ucsb.edu/files/publications/main.pdf
30. Theodoraki EM, Koukouvinos C, Parpoula C. Neural networks for prediction of trauma victims' outcome: Comparison with the TRISS and revised trauma score. Proceedings of the 10th IEEE International Conference on Information Technology and Applications in Biomedicine. Corfu, Greece: IEEE, 2010:1-4.
31. Sefrioui I, Amadini R, Mauro J, El Fallahi A, Gabbrielli M. Survival prediction of trauma patients: a study on US National Trauma Data Bank. Eur J Trauma Emerg Surg. 2017;43(6):805-22.
32. Saleh M, Saatchi R, Burke D. Analysis of the influence of trauma injury factors on the probability of survival. Int J Biol Biomed Eng. 2017;11:88-96.
33. Roveda G, Koledoye MA, Parimbelli E, Holmes JH. Predicting clinical outcomes in patients with traumatic bleeding: a secondary analysis of the CRASH-2 dataset. 2017 IEEE 3rd International Forum on Research and Technologies for Society and Industry (RTSI). Modena, Italy: IEEE, 2017:1-6.
34. Rau CS, Wu SC, Chuang JF, Huang CY, Liu HT, Chien PC, et al. Machine learning models of survival prediction in trauma patients. J Clin Med. 2019;8(6):799.
35. Partridge D, Schetinin V, Li D, Coats TJ, Fieldsend JE, Krzanowski WJ, et al. Interpretability of Bayesian decision trees induced from trauma data. In: Rutkowski L, Tadeusiewicz R, Zadeh LA, Żurada JM, editors. International conference on artificial intelligence and soft computing. Berlin, Heidelberg: Springer; 2006. p. 972-81.
36. Niggli C, Pape HC, Niggli P, Mica L. Validation of a visual-based analytics tool for outcome prediction in polytrauma patients (WATSON trauma pathway explorer) and comparison with the predictive values of TRISS. J Clin Med. 2021;10(10):2115.
37. Wu E, Marthi S, Asaad WF. Predictors of mortality in traumatic intracranial hemorrhage: a national trauma data bank study. Front Neurol. 2020;11:587587.
38. Tsiklidis EJ, Sims C, Sinno T, Diamond SL. Using the National Trauma Data Bank (NTDB) and machine learning to predict trauma patient mortality at admission. PLoS One. 2020;15(11):e0242166.

39. Tran Z, Zhang W, Verma A, Cook A, Kim D, Burruss S, et al. The derivation of an International Classification of Diseases, tenth revision-based trauma-related mortality model using machine learning. J Trauma Acute Care Surg. 2022;92(3):561-6.
40. Schetinin V, Jakaite L, Krzanowski W. Bayesian averaging over decision tree models for trauma severity scoring. Artif Intell Med. 2018;84:139-45.
41. Perkins ZB, Yet B, Sharrock A, Rickard R, Marsh W, Rasmussen TE, et al. Predicting the outcome of limb revascularization in patients with lower-extremity arterial trauma: development and external validation of a supervised machine-learning algorithm to support surgical decisions. Ann Surg. 2020;272(4):564-72.
42. Nemeth C, Amos-Binks A, Burris C, Keeney N, Pinevich Y, Pickering BW, et al. Decision support for tactical combat casualty care using machine learning to detect shock. Mil Med. 2021;186(Suppl 1):273-80.
43. Mou Z, Godat LN, El-Kareh R, Berndtson AE, Doucet JJ, Costantini TW. Electronic health record machine learning model predicts trauma inpatient mortality in real time: a validation study. J Trauma Acute Care Surg. 2022;92(1):74-80.
44. Mossadegh S, He S, Parker P. Bayesian scoring systems for military pelvic and perineal blast injuries: is it time to take a new approach? Mil Med. 2016;181(5 Suppl):127-31.
45. Batchinsky AI, Salinas J, Jones JA, Necsoiu C, Cancio LC. Predicting the need to perform life-saving interventions in trauma patients by using new vital signs and artificial neural networks. In: Combi C, Shahar Y, Abu-Hanna A, editors. Conference on Artificial Intelligence in Medicine in Europe. Berlin, Heidelberg: Springer; 2009. p. 390-4.
46. Bradley M, Dente C, Khatri V, Schobel S, Lisboa F, Shi A, et al. Advanced modeling to predict pneumonia in combat trauma patients. World J Surg. 2020;44(7):2255-62.
47. Liu NT, Holcomb JB, Wade CE, Darrah MI, Salinas J. Utility of vital signs, heart rate variability and complexity, and machine learning for identifying the need for lifesaving interventions in trauma patients. Shock. 2014;42(2):108-14.
48. Liu NT, Holcomb JB, Wade CE, Batchinsky AI, Cancio LC, Darrah MI, et al. Development and validation of a machine learning algorithm and hybrid system to predict the need for life-saving interventions in trauma patients. Med Biol Eng Comput. 2014;52(2):193-203.
49. Kim D, Chae J, Oh Y, Lee J, Kim IY. Automated remote decision-making algorithm as a primary triage system using machine learning techniques. Physiol Meas. 2021;42(2):025006.
50. Larsson A, Berg J, Gellerfors M, Gerdin Warnberg M. The advanced machine learner XGBoost did not reduce prehospital trauma mistriage compared with logistic regression: a simulation study. BMC Med Inform Decis Mak. 2021;21(1):192.

51. Li Y, Wang L, Liu Y, Zhao Y, Fan Y, Yang M, et al. Development and validation of a simplified prehospital triage model using neural network to predict mortality in trauma patients: the ability to follow commands, age, pulse rate, systolic blood pressure and peripheral oxygen saturation (CAPSO) model. Front Med (Lausanne). 2021;8:810195.
52. Scerbo M, Radhakrishnan H, Cotton B, Dua A, Del Junco D, Wade C, et al. Prehospital triage of trauma patients using the random forest computer algorithm. J Surg Res. 2014;187(2):371-6.
53. Zhao Y, Jia L, Jia R, Han H, Feng C, Li X, et al. A new time-window prediction model for traumatic hemorrhagic shock based on interpretable machine learning. Shock. 2022;57(1):48-56.
54. Nemeth C, Pickering B, Amos-Binks A, Harrison A, Pinevich Y, Lowe R, et al. Trauma care decision support under fire. 2019 IEEE International Conference on Systems, Man and Cybernetics (SMC). Bari, Italy: IEEE; 2019. p. 3705-9.
55. Morris R, Karam BS, Zolfaghari EJ, Chen B, Kirsh T, Tourani R, et al. Need for emergent intervention within 6 hours: a novel prediction model for hospital trauma triage. Prehosp Emerg Care. 2022;26(4):556-65.
56. Pennell C, Polet C, Arthur LG, Grewal H, Aronoff S. Risk assessment for intra-abdominal injury following blunt trauma in children: derivation and validation of a machine learning model. J Trauma Acute Care Surg. 2020;89(1):153-9.
57. Paydar S, Parva E, Ghahramani Z, Pourahmad S, Shayan L, Mohammadkarimi V, et al. Do clinical and paraclinical findings have the power to predict critical conditions of injured patients after traumatic injury resuscitation? Using data mining artificial intelligence. Chin J Traumatol. 2021;24(1):48-52.
58. Nederpelt CJ, Mokhtari AK, Alser O, Tsiligkaridis T, Roberts J, Cha M, et al. Development of a field artificial intelligence triage tool: confidence in the prediction of shock, transfusion, and definitive surgical therapy in patients with truncal gunshot wounds. J Trauma Acute Care Surg. 2021;90(6):1054-60.
59. Moulton SL, Mulligan J, Grudic GZ, Convertino VA. Running on empty? The compensatory reserve index. J Trauma Acute Care Surg. 2013;75(6):1053-9.
60. Forsberg JA, Potter BK, Wagner MB, Vickers A, Dente CJ, Kirk AD, et al. Lessons of war: turning data into decisions. EBioMedicine. 2015;2(9):1235-42.
61. Follin A, Jacqmin S, Chhor V, Bellenfant F, Robin S, Guinvarc'h A, et al. Tree-based algorithm for prehospital triage of polytrauma patients. Injury. 2016;47(7):1555-61.
62. Yin JB, Zhao PF, Zhang Y, Han Y, Wang SY. A data augmentation method for war trauma using the war trauma severity score and deep neural networks. Electronics-Switz. 2021;10(21):2657.
63. Feng YN, Xu ZH, Liu JT, Sun XL, Wang DQ, Yu Y. Intelligent prediction of RBC demand in trauma patients using decision tree methods. Mil Med Res. 2021;8(1):33.

64. Lammers D, Marenco C, Morte K, Conner J, Williams J, Bax T, et al. Machine learning for military trauma: novel massive transfusion predictive models in combat zones. J Surg Res. 2022;270:369-75.
65. Johnson MC, Alarhayem A, Convertino V, Carter R 3rd, Chung K, Stewart R, et al. Compensatory reserve index: performance of a novel monitoring technology to identify the bleeding trauma patient. Shock. 2018;49(3):295-300.
66. McLennan JV, Mackway-Jones KC, Smith JE. Prediction of massive blood transfusion in battlefield trauma: development and validation of the Military Acute Severe Haemorrhage (MASH) score. Injury. 2018;49(2):184-90.
67. Mina MJ, Winkler AM, Dente CJ. Let technology do the work: improving prediction of massive transfusion with the aid of a smartphone application. J Trauma Acute Care Surg. 2013;75(4):669-75.
68. Hodgman EI, Cripps MW, Mina MJ, Bulger EM, Schreiber MA, Brasel KJ, et al. External validation of a smartphone app model to predict the need for massive transfusion using five different definitions. J Trauma Acute Care Surg. 2018;84(2):397-402.
69. Yang S, Mackenzie CF, Rock P, Lin C, Floccare D, Scalea T, et al. Comparison of massive and emergency transfusion prediction scoring systems after trauma with a new bleeding risk index score applied in-flight. J Trauma Acute Care Surg. 2021;90(2):268-73.
70. Wei L, Chenggao W, Juan Z, Aiping L. Massive transfusion prediction in patients with multiple trauma by decision tree: a retrospective analysis. Indian J Hematol Blood Transfus. 2021;37(2):302-8.
71. Walczak S. Artificial neural network medical decision support tool: predicting transfusion requirements of ER patients. IEEE Trans Inf Technol Biomed. 2005;9(3):468-74.
72. Shahi N, Shahi AK, Phillips R, Shirek G, Bensard D, Moulton SL. Decision-making in pediatric blunt solid organ injury: a deep learning approach to predict massive transfusion, need for operative management, and mortality risk. J Pediatr Surg. 2021;56(2):379-84.
73. Seheult JN, Anto VP, Farhat N, Stram MN, Spinella PC, Alarcon L, et al. Application of a recursive partitioning decision tree algorithm for the prediction of massive transfusion in civilian trauma: the MTPitt prediction tool. Transfusion. 2019;59(3):953-64.
74. Rickards CA, Vyas N, Ryan KL, Ward KR, Andre D, Hurst GM, et al. Are you bleeding? Validation of a machine-learning algorithm for determination of blood volume status: application to remote triage. J Appl Physiol (1985). 2014;116(5):486-94.
75. Convertino VA, Moulton SL, Grudic GZ, Rickards CA, Hinojosa-Laborde C, Gerhardt RT, et al. Use of advanced machine-learning techniques for noninvasive monitoring of hemorrhage. J Trauma. 2011;71(1 Suppl):S25-32.

76. Davis MA, Rao B, Cedeno PA, Saha A, Zohrabian VM. Machine learning and improved quality metrics in acute intracranial hemorrhage by noncontrast computed tomography. Curr Probl Diagn Radiol. 2022;51(4):556-61.
77. Chen L, McKenna TM, Reisner AT, Gribok A, Reifman J. Decision tool for the early diagnosis of trauma patient hypovolemia. J Biomed Inform. 2008;41(3):469-78.
78. Davuluri P, Wu J, Tang Y, Cockrell CH, Ward KR, Najarian K, et al. Hemorrhage detection and segmentation in traumatic pelvic injuries. Comput Math Methods Med. 2012;2012:898430.
79. Chen L, Reisner AT, McKenna TM, Gribok A, Reifman J. Diagnosis of hemorrhage in a prehospital trauma population using linear and nonlinear multiparameter analysis of vital signs. Annu Int Conf IEEE Eng Med Biol Soc. 2007;2007:3748-51.
80. Lang E, Neuschwander A, Favé G, Abback PS, Esnault P, Geeraerts T, et al. Clinical decision support for severe trauma patients: machine learning based definition of a bundle of care for hemorrhagic shock and traumatic brain injury. J Trauma Acute Care Surg. 2022;92(1):135-43.
81. Reifman J, Chen L, Khitrov MY, Reisner AT. Automated decision-support technologies for prehospital care of trauma casualties. NATO RTO Human Factors & Medicine Panel Symposium: Use of Advanced Technologies and New Procedures in Medical Filed Operators. Essen, Germany; 2010. p. RTO-MP-HFM-182.
82. Zhou Y, Dreizin D, Li Y, Zhang Z, Wang Y, Yuille A. Multi-scale attentional network for multi-focal segmentation of active bleed after pelvic fractures. In: Suk H, Liu M, Yan P, Lian C, editors. International Workshop on Machine learning in Medical Imaging. Cham: Springer; 2019. p. 461-9.
83. Ginat D. Analysis of head CT scans flagged by deep learning software for acute intracranial hemorrhage. Neuroradiology. 2020;62(3):335-40.
84. Ginat D. Implementation of machine learning software on the radiology worklist decreases scan view delay for the detection of intracranial hemorrhage on CT. Brain Sci. 2021;11(7):832.
85. Zeineddin A, Hu P, Yang S, Floccare D, Lin CY, Scalea TM, et al. Prehospital continuous vital signs predict need for resuscitative endovascular balloon occlusion of the aorta and resuscitative thoracotomy prehospital continuous vital signs predict resuscitative endovascular balloon occlusion of the aorta. J Trauma Acute Care Surg. 2021;91(5):798-802.
86. Chapman MP, Moore EE, Burneikis D, Moore HB, Gonzalez E, Anderson KC, et al. Thrombelastographic pattern recognition in renal disease and trauma. J Surg Res. 2015;194(1):1-7.
87. Li K, Wu H, Pan F, Chen L, Feng C, Liu Y, et al. A machine learning-based model to predict acute traumatic coagulopathy in trauma patients upon emergency hospitalization. Clin Appl Thromb Hemost. 2020;26:1076029619897827.

88. He L, Luo L, Hou X, Liao D, Liu R, Ouyang C, et al. Predicting venous thromboembolism in hospitalized trauma patients: a combination of the caprini score and data-driven machine learning model. BMC Emerg Med. 2021;21(1):60.
89. Perkins ZB, Yet B, Marsden M, Glasgow S, Marsh W, Davenport R, et al. Early identification of trauma-induced coagulopathy: development and validation of a multivariable risk prediction model. Ann Surg. 2021;274(6):e1119-e1128.
